# Multi-omics profiling reveals single-seed mutants of *Ephedra saxatilis* as dominant variants in high-altitude Xizang

**DOI:** 10.1186/s12870-025-07153-x

**Published:** 2025-08-22

**Authors:** Mengnan Lu, Shiyan Wang, Yonghong Zhou, Xiaona Wang, Hao Su, Yanbing Gong, Ji De

**Affiliations:** 1 Key Laboratory of Biodiversity and Environment on the Qinghai-Tibetan Plateau, Ministry of Education, School of Ecology and environment, Xizang University, Lhasa, 850000 China; 2 School of Ecology and environment, Xizang University, Lhasa, Xizang China; 3 College of Life Sciences, Wuhan University, Wuhan, China

**Keywords:** *Ephedra*, Mutant seed, Metabolism, Hormone, Gene expression, Characterization

## Abstract

*Ephedra* species, important Tibetan medicinal plants, are widely distributed across the Qinghai-Tibet Plateau at altitudes of 2700–5000 m. Their adaptation to high-altitude environments, such as low temperatures, strong UV radiation and low oxygen, is still poorly understood. This study investigated the morphological, metabolic, and genetic mechanisms underlying the reproductive advantage of a unique single-seed variant observed in high-germination-rate *Ephedra* species. Seeds from six *Ephedra* species were collected for germination assays and electron microscopic analysis. Results showed that *E. saxatilis*, *E. intermedia*, and *E. monosperma* exhibited significantly higher germination rates (Germination rates > 65%) and predominantly produced single-seed variants, while others mainly produced double seeds. Analysis of burr and fold numbers of phenotypic traits showed a significant positive correlation with germination rates. Time-course metabolomics analysis identified 762 KEGG annotated metabolites, and revealed *E. saxatilis* as the dominant species due to its faster metabolic rate, particularly simulated high-altitude conditions. Absolute hormone quantification highlighted the single-seed variant of *E. saxatilis* as the dominant type, with ABA content peaking in the shed seed coat. ABA exhibited antagonistic interactions with 2MeScZR, SA, IAA, GA7, IPR, and t-CA, suggesting a complex hormonal regulation network. Co-expression network analysis integrating transcriptome and hormone data predicted 23 key genes regulating seed germination adaptation. This study provides novel insights into the ecological and evolutionary significance of single-seed variation in high-altitude adaptation. The findings have potential applications in high-altitude plant breeding, conservation, and sustainable utilization of *Ephedra* species. Future research should focus on the genetic basis of single-seed variation and its role in other high-altitude plant species.

**Supplementary Information:**

The online version contains supplementary material available at 10.1186/s12870-025-07153-x.

## Introduction

*Ephedra*, a renowned Chinese herbal medicine, has been widely used for centuries due to its well-defined medicinal components, such as ephedrine and pseudoephedrine [1–3]. Notably, the content of these bioactive compounds varies significantly with environmental conditions [3–7], highlighting the importance of studying *Ephedra* in diverse habitats. Beyond its medicinal value, *Ephedra* plays a critical ecological role in preventing soil erosion, owing to its extensive root systems [7–9]. With over 60 species identified globally [10], *Ephedra* exhibits remarkable diversity and adaptability. However, only seven species, including *Ephedra. saxatilis*, *E. gerardiana*, *E. minuta*, *E. intermedia*, *E. saxatilis* var. *mairei*, *E. likiangensis*, and *E. monosperma* are found in Xizang (Tibet) [11, 12]. In contrast, the dominant species in China’s plains, such as *Ephedra. sinica* and *Ephedra. intermedia*, have been extensively exploited for medicinal purposes, windbreak and sand fixation [1, 13]. Although *Ephedra* has significant ecological and medicinal value, due to the high altitude in the Xizang, the distribution of *Ephedra* is relatively scarce. Therefore, identifying and screening the dominant species of *Ephedra* that are adapted to the high-altitude environment is of vital importance for achieving sustainable cultivation, providing a stable source of medicinal resources, and offering effective strategies for ecological restoration in Xizang.

In Xizang, *Ephedra* species are primarily distributed at altitudes ranging from 2800 to 5000 m [2, 3], where they endure extreme environmental stresses such as prolonged drought, low temperatures, and strong ultraviolet radiation [13]. These harsh conditions result in lower *Ephedra* coverage compared to low altitude regions [14], underscoring the need to identify and cultivate high-altitude-adapted species to improve coverage rates. During seed collection in Xizang, we observed a unique phenomenon: the presence of single seeds, which have rarely been reported in previous studies. These cones with varying numbers of seeds, including single seed, double seed, and triple seed, potentially reflecting adaptive strategies to high-altitude stresses. This might be due to the influence of high altitude, temperature and ultraviolet rays, which cause changes in the quantity in the cones [15]. In order to explore the adaptation mechanism of *Ephedra* seeds in high-altitude environments, this study adopted a multidisciplinary approach, combining germination tests, transcriptomics, metabolomics and hormone quantification, to identify *Ephedra* seeds with growth advantages under high-altitude stress conditions.

Seed metabolism is a critical determinant of seed survival and adaptation [16]. Primary metabolites, such as carbohydrates and amino acids provides energy necessary for seed germination and subsequent growth [17–19]. Metabolic rate, along with phenotypic traits like seed weight and size, can serve as indicators of seed quality and adaptability. Furthermore, plant hormones, despite their low concentrations, play pivotal roles in regulating seed development and germination [20–23]. The changes in internal hormones can be used to screen for mutant seeds and normal seeds in the selected *Ephedra*. Key hormones, including auxin, cytokinin, gibberellin, and abscisic acid, orchestrate complex physiological processes during plant growth [24–26]. By analyzing hormonal changes, we can distinguish between single seeds and normal seeds, elucidate the mechanisms underlying their differences, and identify hormonal markers associated with high-altitude adaptation.

In this study, seeds from six *Ephedra* species in Xizang were collected and identified to distinct seed variants, including single-seed, double-seed, and triple-seed types within individual cones. Our objectives are threefold: (1) to compare the germination rates and growth performance of single seeds and normal seeds, identifying those with the highest vitality; (2) to use metabolomics and phenotypic characterization to select high-quality seeds; and (3) to construct hormone regulatory networks through transcriptomics and absolute hormone quantification, elucidating the mechanisms driving hormonal changes and seed adaptation. By distinguishing between single seeds and normal seeds, we aim to clarify the advantages of single seeds in high-altitude environments. This research will provide a theoretical foundation for the development and utilization of *Ephedra* germplasm resources, supporting large-scale afforestation, soil and water conservation, and sustainable medicinal resource production in Xizang.

## Materials and methods

### Plant materials and resource distribution

**Fig. 1:**
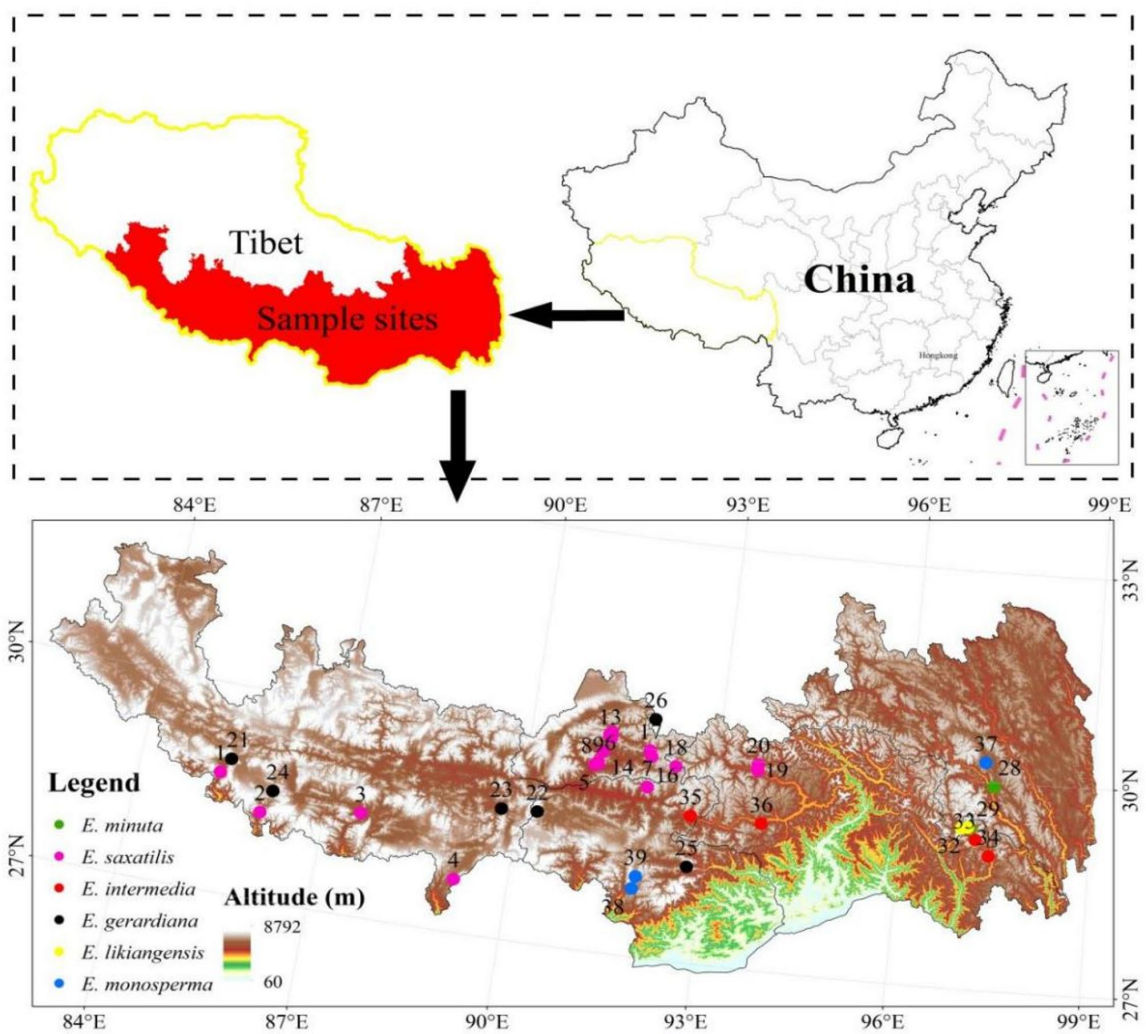
Distribution map of six high-altitude *Ephedra* species in Xizang. Note: Including *Ephedra. saxatilis*, *Ephedra. gerardiana*, *Ephedra. minuta*, *Ephedra. intermedia*, *Ephedra. likiangensis*, *Ephedra. monosperma.* According to local regulations, Tibet renamed to Xizang

The high-altitude *Ephedra* samples were primarily collected from the southern region of Xizang (Tibet), as illustrated in Fig. 1. All collected samples and specimens are currently stored at the herbarium of College of Ecology and Environment, Xizang University. Species identification was performed by Professor Ji De based on the morphological description in Flora of China. The voucher specimen numbers are as follows: *Ephedra. Saxatilis*: TUE20240806S1-JD; *Ephedra. Gerardiana*: TUE20240831G2-JD; *Ephedra. Likiangensis*: TUE20240901L3-JD; *Ephedra. Intermedia*: TUE20240817I4-JD; *Ephedra. Minuta*: TUE20240824M5-JD; *Ephedra. Monosperma*: TUE20240925MO6-JD. During the collection of *Ephedra*, a total of six *Ephedra* species of were identified across 39 sampling points. The distribution of sampling points is as follows: 20 sampling points of *E. saxatilis*, 6 sampling points of *E. gerardiana*, 3 sampling points of *E. minuta*, 3 sampling points of *E. likiangensis*, 4 sampling points of *E. intermedia*, and 3 sampling points of *E. monosperma.* Detailed geographic coordinates (longitude, latitude, and altitude) of the sampling points are provided in Table S1. Seeds from *E. saxatilis*, *E. gerardiana*, *E. minuta*, *E. likiangensis*, *E. intermedia* and *E. monosperma* collected for subsequent experiments, including germination assays, morphological characterization, metabolomic analysis, and transcriptomic profiling.

### Morphological observation and germination

#### Morphological characterization

For *E. saxatilis*, *E. gerardiana*, *E. minuta*, *E. likiangensis*, *E. intermedia* and *E. monosperma*, 100 fully mature and healthy seeds were selected for morphological observation and germination experiments. Seed preparation for light microscopy (LM) followed the protocol described by Leslie et al. [27]. Seed morphology was analyzed using an ultra-depth-of-field microscope (DM-2700, Leica Microsystems, Germany) at magnifications of 50× and 200×. The following morphological traits were recorded and measured: seed length and width were measured using ImageJ software (National Institutes of Health, USA); seed shape was were conducted following a modified protocol from Marcel et al. [27] and Lu et al. [28]; the color of the seed is determined using a colorimetric card under standardized lighting conditions (for example, brown, black, or gray); the shape and color were determined following result; the length and width of seeds were measured using the image analysis software ImageJ [29]. Additionally, the weight of 100 seeds was calculated by weighing three replicates of 100 seeds each and averaging the results.

#### Germination assay

Seed germination tests were conducted following a modified protocol from Rathee et al. [30]. Briefly, seeds were surface-sterilized by soaking in a 1% sodium hypochlorite solution for 10 min, followed by three rinses with distilled water. For each taxon, 50 seeds were plated in triplicate on sterilized filter paper or agar medium and incubated at 25 °C under controlled light conditions (12-hour light-dark cycle) for 10 days. Germination was defined as the emergence of the radicle from the seed coat. The number of germinated seeds was recorded daily, and the germination rate was calculated as follows [28]:

$$\begin{aligned}\mathrm{Germination}\;\mathrm{Rate}\;(\%)\;=&(\mathrm{Germinated}\;\mathrm{Seeds}\\&/\mathrm{Total}\;\mathrm{of}\;\mathrm{Test}\;\mathrm{Seeds})\times\;100\% \end{aligned}$$

Seeds that germinated within 24, 48, and 72 h were selected for subsequent experiments, including metabolomic and transcriptomic analyses.

#### Scanning electron microscopy

For SEM analyses, 10 seeds from *E. saxatilis*, *E. gerardiana*, *E. minuta*, *E. likiangensis*, *E. intermedia* and *E. monosperma* were prepared and examined. Seeds were transferred into aluminum stubs using double-sided adhesive tape [31]. Prior to imaging, samples were sputter-coated with a thin layer of gold to enhance conductivity. Whole seed surface to local scanning from 400 μm to 10 μm, the morphological features and surface ornamentation of seed epidermal cells were observed and recorded using a Guoyi 5000 Pro scanning electron microscope (Guoyi Quantum AG, Hefei, China) at an accelerated voltage of 7 kV. Six points were selected 50 μm apart to observe the surface texture, and the four points showed the same texture, which represented the surface structure of the seed.

### Metabolomics analysis

#### Sample processing

Because seeds of *E. saxatilis*, *E. intermedia* and *E. monosperma* have higher germination rate, single and double seeds from *E. saxatilis*, *E. intermedia* and *E. monosperma* that germinated at 24, 48, and 72 h were selected for metabolomic analysis. All experiments were conducted at Xizang University. Seed samples were freeze-dried and ground into a fine powder using a mortar and pestle under liquid nitrogen. Approximately 100 mg of seed tissue was homogenized in prechilled 80% methanol and vortexed thoroughly. The homogenate was incubated on ice for 5 min and then centrifuged at 15,000 g and 4 °C for 20 min. The supernatant was collected and diluted to a final concentration of 53% methanol using UHPLC-MS grade water. The diluted samples were transferred to fresh Eppendorf tubes and centrifuged again at 15,000 g and 4 °C for 20 min. The final supernatant was filtered through a 0.22 μm membrane and injected into the UHPLC-MS/MS system for analysis.

#### UHPLC-MS/MS analysis

Analysis of metabolites in the seeds of *E. saxatilis*, *E. intermedia* and *E. monosperma* by Ren [32] and Wang [33] with some modifications. UHPLC-MS/MS analyses were performed using a Vanquish UHPLC system (ThermoFisher, Germany) coupled with an Orbitrap Q ExactiveT^M^ HF mass spectrometer or Orbitrap Q Exactive^TM^HF-X mass spectrometer (Thermo Fisher, Germany) in Novogene Co., Ltd. (Beijing, China). Samples were injected onto aHypersil Goldcolumn (100 × 2.1 mm, 1.9 μm) using a 12-min linear gradient at a flowrate of 0.2 mL/min. The eluents for the positive and negative polarity modes were eluent A (0.1%FA in Water) and eluent B (Methanol). The solvent gradient was set as follows: 2% B, 1.5 min; 2–85% B, 3 min; 85–100% B, 10 min; 100-2% B, 10.1 min; 2% B, 12 min. Q Exactive^TM^HF mass spectrometer was operated in positive/negative polarity mode with spray voltage of 3.5 kV, capillary temperature of 320℃, sheath gas flow rate of 35 psi and aux gas flow rate of 10 L/min, S-lens RF level of 60, Aux gas heater temperature of 350℃.

The data acquisition instrument system mainly includes Ultra Performance Liquid Chromatography (UPLC) (ExionLC™ AD), https://sciex.com.cn/) and Tandem Mass Spectrometry (MS/MS) (QTRAP^®^ 6500+, https://sciex.com.cn/).

#### Absolute quantitative analysis of hormones

According to the above experiments, *E. saxatilis* seeds were screened to have a relatively high metabolic rate, so single and double seeds of *E. saxatilis* were selected for absolute quantification. Absolute quantitative analysis of hormones was commissioned by Metware Biotechnology Co., LTD. (Wuhan, China). Absolute quantitative of hormones was performed on seeds with longer germination time. The seeds were 24, 48, 72, 96 and 120 h, respectively. Preparation of 0.01 ng/mL, 0.05 ng/mL, 0.1 ng/mL, 0.5 ng/mL, 1 ng/mL, 5 ng/mL, 10 ng/mL, 50 ng/mL, 100 ng/mL, 200 ng/mL, 500 ng/mL of different concentrations of hormone standard solution. The mass spectrum peak intensity data of the corresponding quantitative signal of each concentration standard were obtained. The Concentration Ratio between external standard and internal standard is the horizontal coordinate, and the peak Area Ratio between external standard and internal standard is the longitudinal coordinate, and the standard curve of different substances is drawn. Standard curve of self-established database of 109 hormones shown in Table S2. The absolute quantitative of *Ephedra* seed hormones comparison database was carried out.

### Transcriptomic analysis

All seeds with absolute hormone quantification were selected for transcriptomic analysis, the seeds were 24, 48, 72, 96 and 120 h, respectively. Transcriptome sequencing was commissioned by Metware Biotechnology Co., LTD. (Wuhan, China) to extract RNA from 30 samples. Qubit 4.0 fluorometer/MD Microplate reader (Shanghai, China) and Qsep400 bioanalyzer (Shanghai, China) were used. The cDNA library was sequenced by Illumina high throughput sequencing platform using SBS technology. The resulting images are converted by CASAVA into a large amount of high-quality sequencing data. The raw sequencing data was then filtered using fastp software (v0.23.4). The transcriptional sequence of the species was obtained by using Trinity assembly. With that, transcriptional sequence was processed to remove the redundancy and obtain the Unigene sequence. High-quality read and de-redundant transcripts identify differentially expressed genes in different samples. They were compared with databases such as KEGG, NR, Swiss-Prot, GO, KOG and Trembl. This process provides valuable functional information about genes. HMMER software (3.3.2) was used to analyze the amino acid sequences of Unigenes and align them with the Pfam database, which further enriched the knowledge of protein families and the domains in the samples. Together, this comprehensive pipeline of RNA sequencing and analysis provides a powerful tool for studying gene expression and functional signatures in different species.

### Statistical analysis

These metabolites were annotated using the KEGG database (https://www.genome.jp/kegg/pathway.html), HMDB database (https://hmdb.ca/metabolites) and LIPID Maps database(http://www.lipidmaps.org). Principal components analysis (PCA) and Partial least squares discriminant analysis (PLS-DA) were performed at metaX (a flexible and comprehensive software for processing metabolomics data). The data were analyzed through the free online platform of the majorbio cloud platform (https://magic-plus.novogene.com/).

We applied univariate analysis (t-test) to calculate the statistical significance (P-value). The metabolites with VIP > 1 and P-value < 0.05 and fold change ≥ 2 or FC ≤ 0.5 were considered to be differential metabolites. Volcano plots were used to filter metabolites of interest which based on log2(Fold Change) and -log10(p-value) of metabolites by ggplot2 in R language. All experiments were conducted using three independent biological replicates. Statistical analysis was conducted using limma software in the R statistical package (http://www.rproject.org), with analysis of ANOVA and Duncan’s multiple range test. *p* < 0.05 was considered statistically significant.

## Results

### Physiology and characterization of six kinds of *Ephedra* seeds

**Fig. 2:**
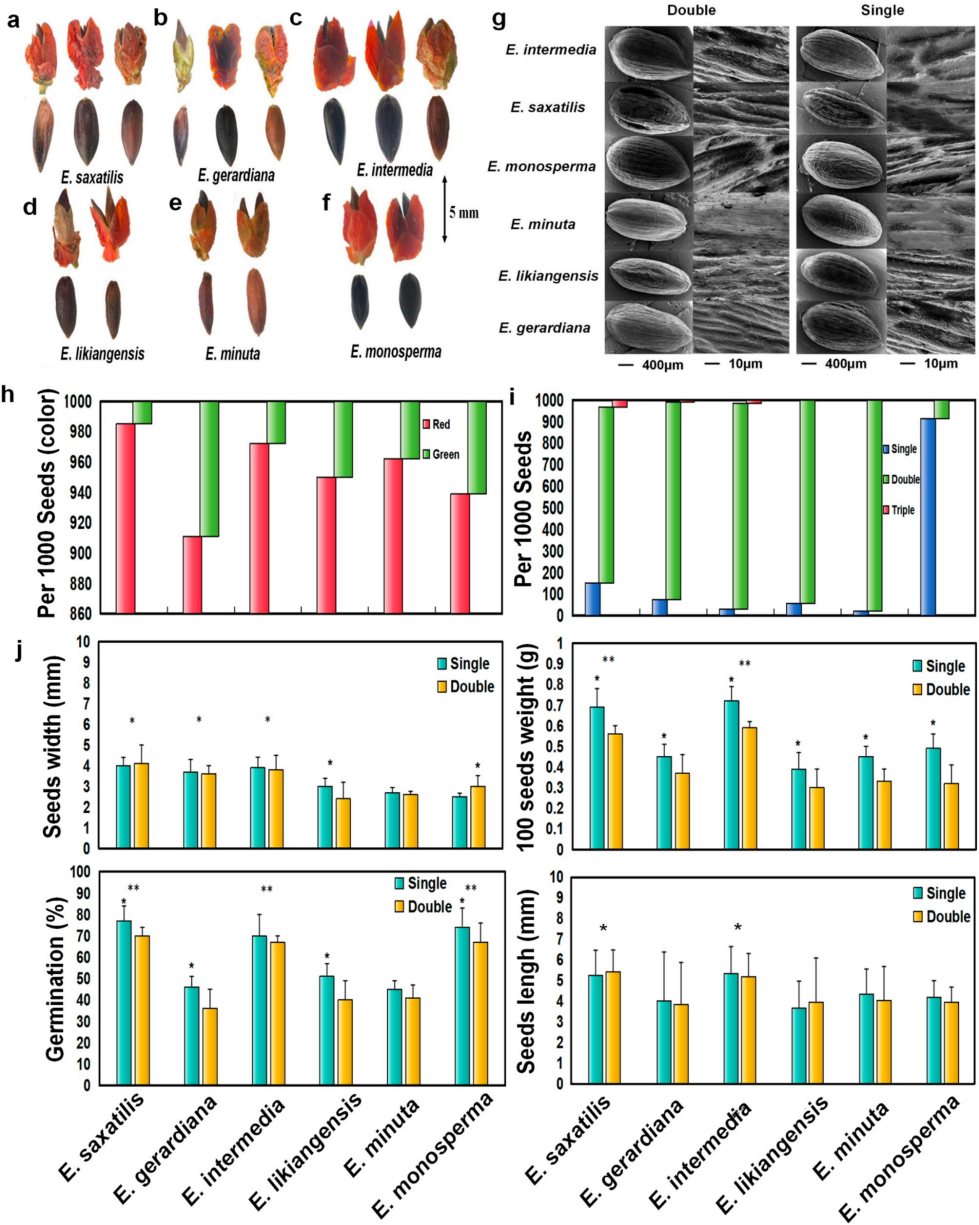
Morphology and physiological indicators of different species of *Ephedra* seeds. **a-f** Morphological diversity of *Ephedra* seeds. **g** Scanning electron microscope (SEM) images highlighting surface ultrastructure variations. **h** Color variation of cones among species, ranging from green to reddish-brown.**i** Number of seeds per cone, showing significant inter specific variation. **j** Comparative analysis of seed width, weight, germination rate, and length: Seed width and length were measured using digital calipers, weight was determined using a precision balance, and germination rates were calculated under controlled conditions (25 °C, 12-hour light-dark cycle). Data are presented as Mean ± SD (*n* = 3). (**p < 0.01; *p < 0.05)

As illustrated in Fig. 2, the seeds of the six *Ephedra* species exhibited distinct surface morphological differences. *E. saxatilis* (a), *E. gerardiana* (b), and *E. intermedia* (c) produced cones containing single, double, and triple seeds, whereas *E. likiangensis* (d), *E. minuta* (e), and *E. monosperma* (f) produced cones with only single and double seeds. Based on SEM, distinct structural types were identified: single seeds of all six species generally featured more small burrs compared to double or triple seeds. Among double seeds, *E. saxatilis*, *E. monosperma*, and *E. intermedia* exhibited more burrs, while *E. gerardiana*, *E. likiangensis*, and *E. minuta* had fewer burrs (g), there was a significant positive correlation between these burrs and germination rate (*r* > 0.8; *p* < 0.05). The color of bracts of all *Ephedra* species is red more than green (Fig. 2h). The proportions of single, double, and triple seeds varied among species (Fig. 2i): *E. saxatilis*: 15.1% single, 81.6% double, and 3.3% triple seeds. *E. gerardiana*: 7.4% single, 91.6% double, and 1.0% triple seeds. *E. intermedia*: 3.1% single, 95.4% double, and 1.5% triple seeds. *E. likiangensis*: 5.7% single and 94.3% double seeds. *E. minuta*: 2.2% single and 97.8% double seeds. *E. monosperma*: 91.3% single and 8.7% double seeds.

Germination rates varied significantly among seed types and species (Fig. 2j). Single seeds consistently exhibited higher germination rates than double seeds. The overall germination rates of *E. saxatilis*, *E. intermedia*, and *E. monosperma* were significantly higher than those of the other species, with *E. saxatilis* single seeds achieving the highest germination rate of 77.4 ± 7.1%. In terms of seed dimensions, the average width of *E. saxatilis*, *E. gerardiana*, and *E. intermedia* seeds was significantly greater than that of the other species. *E. saxatilis* and *E. intermedia* seeds were significantly longer than those of the other species. The weight of 100 single seeds was consistently higher than that of 100 double seeds across all species. *E. saxatilis*, *E. intermedia*, and *E. monosperma* were selected for further experiments due to their higher germination rates and distinct morphological characteristics.

### Metabolic analysis of three species of *Ephedra* seeds

**Fig. 3:**
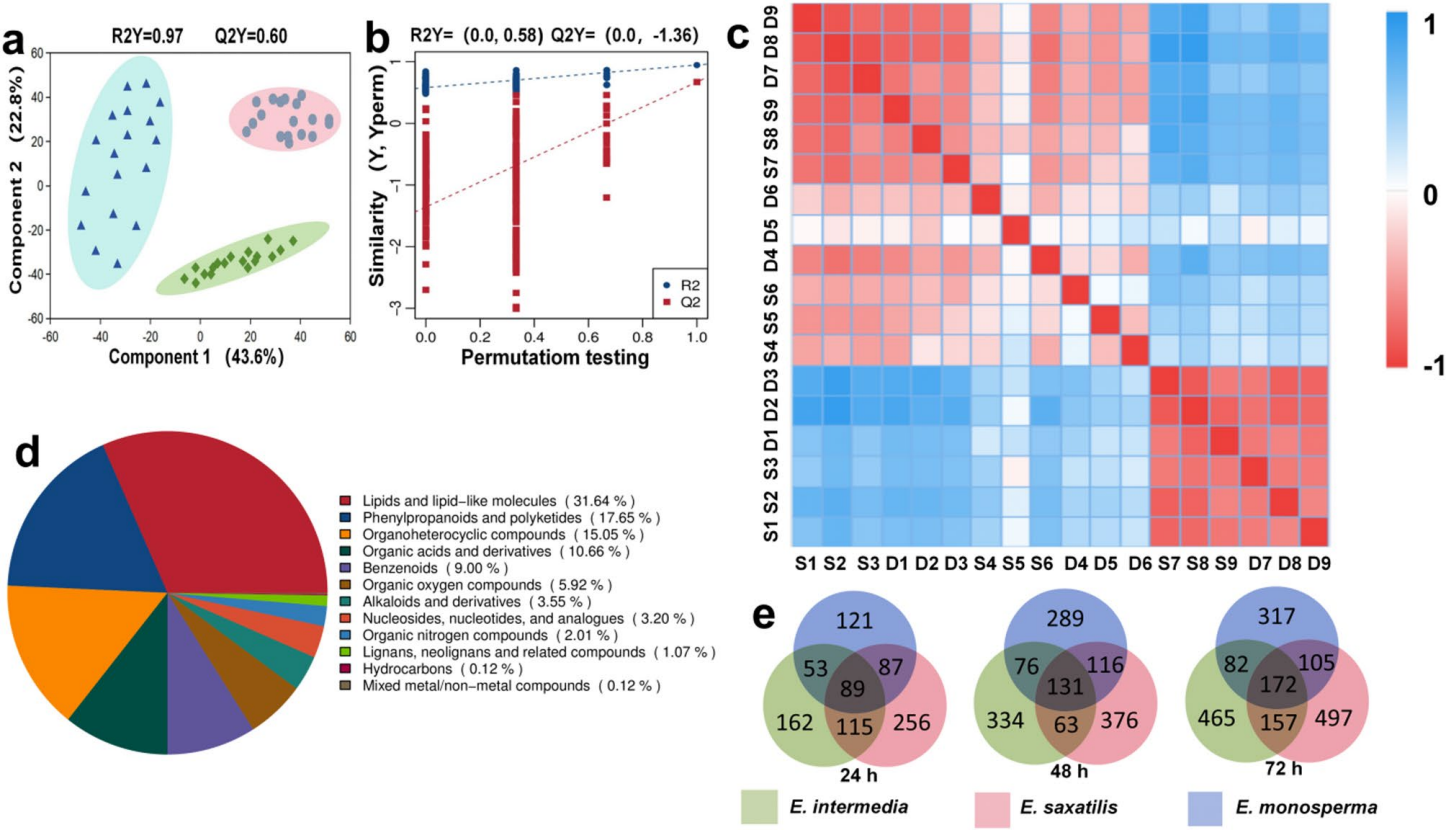
Metabolic profiling and analysis of *Ephedra* seeds at different germination stages. **a** Partial least squares-discriminant analysis (PLS-DA) of *Ephedra* seeds at three germination time points (24 h, 48 h, and 72 h). The model demonstrates clear separation among the three stages, with R2Y = 0.97 and Q2 = 0.60, indicating significant metabolic differences (*p* < 0.05). **b** Validation of the PLS-DA model using a 200-response permutation test (RPT). R2 represented the sum of variances that could be explained by the model. Q2 represents the predictive ability of the model. When using the RPT test, Q2 is required to be less than zero. **c** Heatmap showing the correlation matrix of metabolic profiles among samples. **d** Classification of metabolites annotated in the HUMB database. A total of 894 metabolites were identified and grouped into 12 categories. **e** Venn diagram illustrating the overlap and uniqueness of metabolites detected at different germination stages (24 h, 48 h, and 72 h). The number of metabolites increased progressively over time, with the highest complexity observed at 72 h

We conducted a comprehensive metabolomic analysis on three species: *E. intermedia*, *E. monosperma*, and *E. saxatilis*. As shown in Fig. 3a, partial least squares-discriminant analysis (PLS-DA) was performed to assess metabolic differences among the three stages. In our analysis, R2Y = 0.97 and Q2 = 0.60, suggesting significant metabolic differences among the three stages (*p* < 0.05). To prevent model overfitting, a 200-response permutation test (RPT) was conducted (Fig. 3b). Cluster analysis of the detected metabolites from 18 samples (Fig. 3c) revealed distinct metabolic patterns among the three *Ephedra* species. Samples were labeled as S (single seed) and D (double seed). The metabolites of *E. intermedia* (S1-D3) showed similarity within the species but differed significantly from those of *E. monosperma* (S4-D6). Notably, *E. saxatilis* (S7-D9) exhibited a unique metabolic composition, distinct from the other two species. Additionally, single-seed and double-seed variants within each species showed consistent metabolic differences.

A total of 894 metabolites were annotated using the HUMB database (Fig. 3d). These metabolites were classified into 12 categories, with lipids being the most abundant (31.64%), followed by phenylpropanoids (17.65%) and organoheterocyclic compounds (15.05%). Venn diagram analysis (Fig. 3e) revealed that the number of metabolites increased progressively over time (24 h, 48 h, and 72 h) for all three species. By 72 h, the metabolic profiles reached their highest complexity, suggesting active metabolic reprogramming during seed development.

### Analysis of metabolic pathways in three species of *Ephedra* seeds

**Fig. 4:**
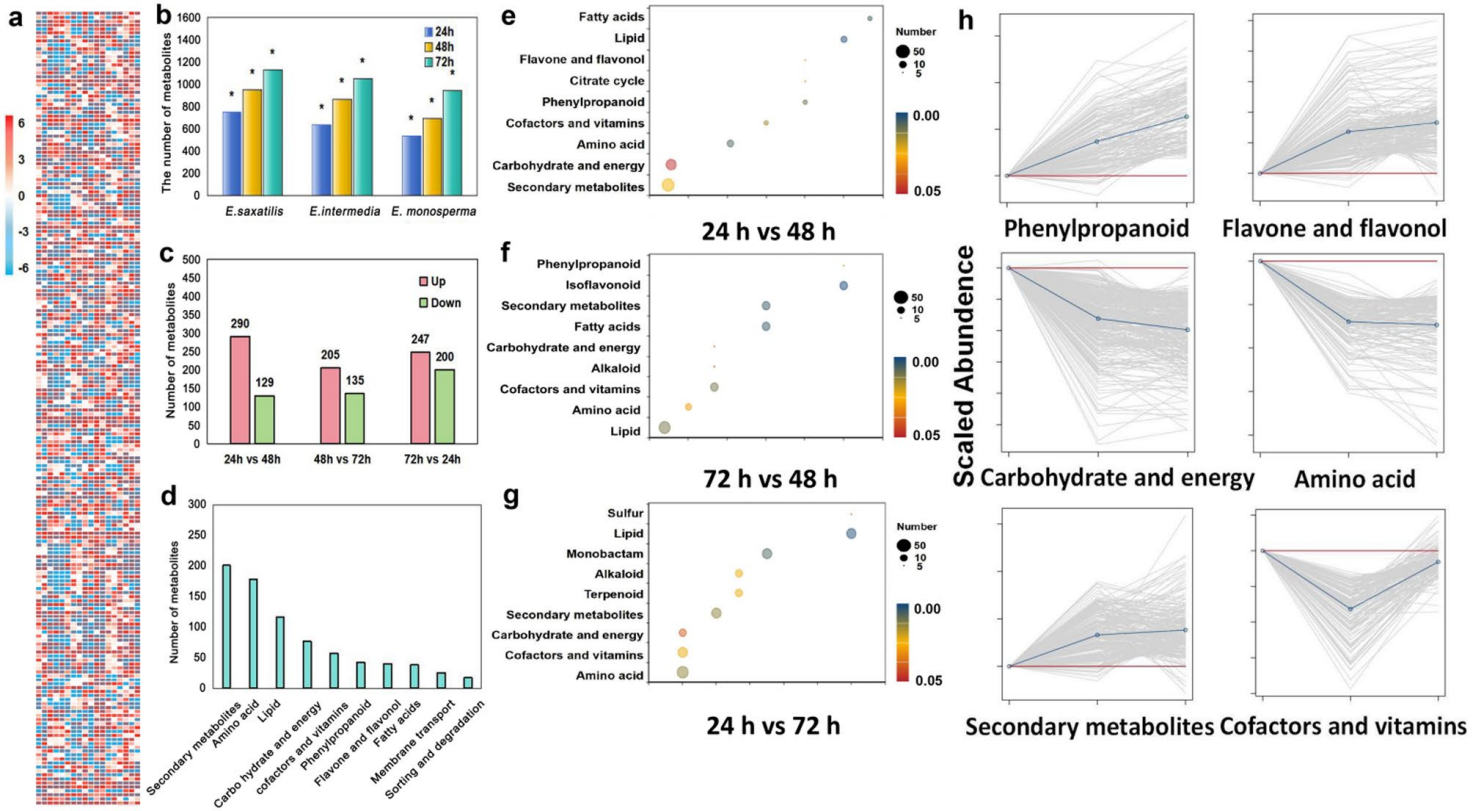
Metabolic profiling and pathway analysis of *Ephedra* seeds during germination. **a** Cluster analysis of metabolites from three *Ephedra* species (*E. saxatilis*, *E. intermedia*, and *E. monosperma*) at three germination stages (24 h, 48 h, and 72 h).) Cluster analysis of metabolites from three *Ephedra* species (*E. saxatilis*, *E. intermedia*, and *E. monosperma*) at three germination stages (24 h, 48 h, and 72 h). **b** Dynamic changes in metabolite quantities during germination. The number of metabolites increased significantly over time, with *E. saxatilis* showing the highest metabolic complexity. (**p < 0.01; *p < 0.05) (**c**) Comparison of upregulated and downregulated metabolites at different germination stages. Most metabolites showed increased abundance over time, while a smaller subset decreased. **d** KEGG-annotated metabolic pathways. **e-f** Bubble plots of differential metabolites in KEGG pathways at 24 h, 48 h, and 72 h. The size of the bubbles represents the number of differential metabolites, and the color indicates the significance of the differences (*p* < 0.05). **h** Kinetic analysis of metabolites in six key pathways. The gray lines represent the relative enrichment of metabolites in different samples, the blue line shows the average enrichment of all metabolites in the pathway, and the red line represents the enrichment of the Y-axis contro

To elucidate the metabolic dynamics during seed germination, we conducted a comprehensive analysis of metabolic pathways in three *Ephedra* species: *E. saxatilis*, *E. intermedia*, and *E. monosperma*. As shown in Fig. 4a, the number of metabolites detected in *E. saxatilis* seeds increased significantly over time (Fig. 4b), with 751 metabolites at 24 h, 949 at 48 h, and 1131 at 72 h. This trend suggests active metabolic reprogramming during seed germination. Comparative analysis of all treatment stages (Fig. 4c) further revealed that the relative content of most metabolites increased over time, while a smaller subset decreased. This indicates that prolonged treatment time enhances the overall metabolic activity in *Ephedra* seeds.

A total of 762 metabolites were annotated using the KEGG database (Fig. 4d). These metabolites were primarily enriched in the top 3 metabolic pathways, with secondary metabolism (201 metabolites), with amino acid metabolism (178 metabolites) and lipid metabolism (116 metabolites). To identify stage-specific metabolic changes, differential metabolites were compared across treatment times. The size of circles in Fig. 4e-g represents the number of differential metabolites in each pathway. Secondary metabolism, carbohydrate and energy metabolism, and amino acid metabolism exhibited the highest number of differential metabolites. Trend analysis of metabolites across all pathways revealed significant changes in six key pathways (Fig. 4h). Phenylpropanoid, flavone and flavonol biosynthesis: Metabolites in these pathways showed a consistent upward trend, indicating their potential role in stress response and antioxidant activity.

### Metabolic dynamics and Species-Specific adaptations in *Ephedra* seeds

**Fig. 5:**
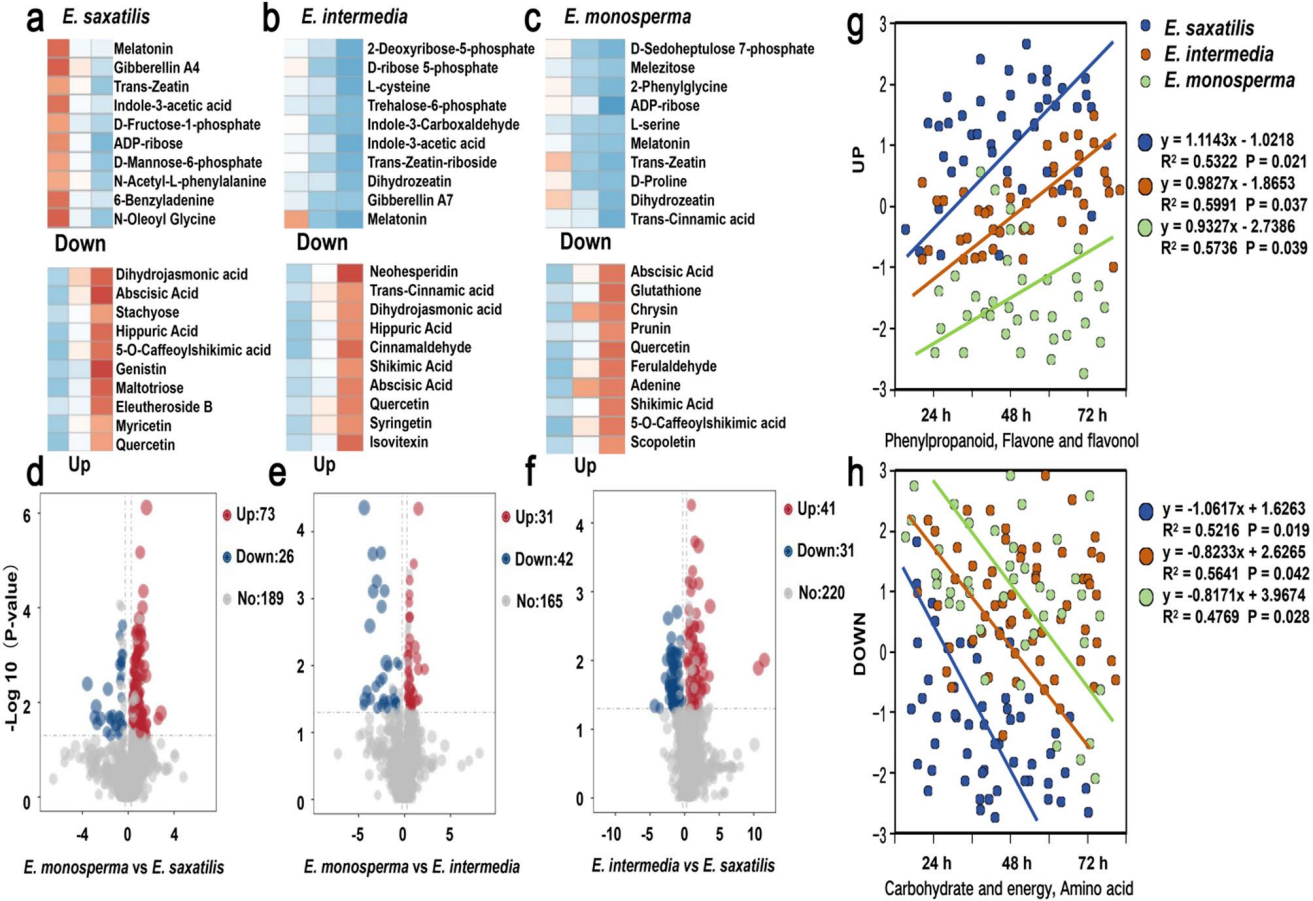
Differential metabolite analysis and pathway-specific changes in *Ephedra* species during germination. **a-c** Top 10 differential metabolites in three species in *Ephedra*. The heatmap reveals distinct metabolic dynamics compared in three species in *Ephedra.* **d-f** Volcano plots of relative content changes in metabolites across the three *Ephedra* species. The x-axis represents the fold change (log2), and the y-axis represents the statistical significance (-log10 p-value). Colored circles indicate metabolites with significant increases (red) or decreases (blue) in relative content (*p* < 0.05). **g** Rate of increase in metabolites within the phenylpropanoid, flavone, and flavonol pathways for the three *Ephedra* species. *E. saxatilis* exhibited the fastest increase. **h** Rate of decrease in metabolites within the carbohydrate and energy metabolism and amino acid pathways in three *Ephedra* species. *E. saxatilis* showed the most rapid decline

After analyzing the top 10 downregulated metabolites across all *Ephedra* species (Fig. 5a-c), we identified a consistent decrease in key metabolites, including 2-Deoxyribose-5-phosphate, gibberellins, trans-Zeatin, melatonin, ADP-ribose, indole-3-carboxylic acid, and indole-3-acetic acid and so on. These metabolites were categorized into three functional groups: energy metabolism, such as 2-Deoxyribose-5-phosphate, ADP-ribose, D-Fructose-1-phosphate, Trehalose-6-phosphate and so on; growth hormones, such as gibberellins, trans-Zeatin, indole-3-acetic acid, indole-3-carboxylic acid, melatonin and so on; amino acid derivatives such as N-Acetyl-L-phenylalanine, 6-Benzyladenine, N-Oleoyl-Glycine and so on. In contrast, the top 10 upregulated metabolites included abscisic acid, quercetin, mannose, myricetin, apigenin, maltotriose and so on. These metabolites were classified into monosaccharides, such as mannose, maltotriose, stachyose, stachyose and so on; growth hormones, such as dihydrojasmonic acid, ABA and so on; Flavonoids, such as myricetin, quercetin, apigenin, syringetin and so on.

Comparative analysis of differential metabolites among the three *Ephedra* species revealed distinct metabolic patterns (Fig. 5d-f). *E. saxatilis* exhibited a higher proportion of upregulated metabolites compared to downregulated metabolites, indicating more vigorous metabolic activity and dynamic changes during germination.

To further investigate species-specific metabolic adaptations, we analyzed the relative content changes in key pathways (Fig. 5g-h): Carbohydrate and Energy Metabolism: The relative content of metabolites in *E. saxatilis* decreased the fastest among the three species. Amino Acid Metabolism: Similarly, *E. saxatilis* showed the most rapid decline in amino acid-related metabolites. Phenylpropanoid and Flavonoid Biosynthesis: In contrast, E. *saxatilis* exhibited the fastest increase in metabolites within the phenylpropanoid and flavonoid pathways.

### Hormone dynamics in single and double seeds of *E. saxatilis*

**Fig. 6:**
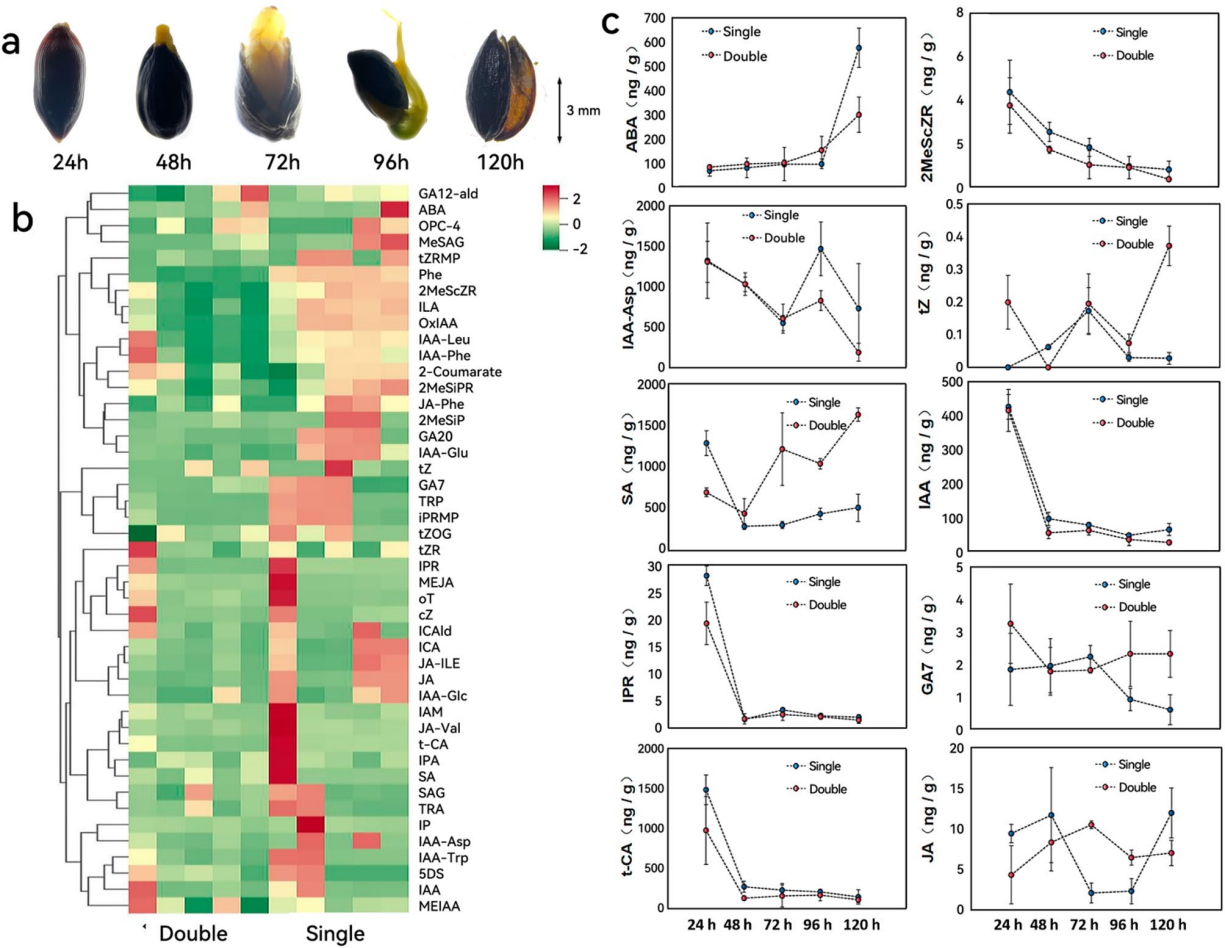
Hormone quantification and dynamics during seed germination in *E. saxatilis. ***a** Germination dynamics of E. saxatilis seeds at five time points (24 h, 48 h, 72 h, 96 h, and 120 h). **b** Cluster heatmap of 45 hormones showing significant differences in abundance across germination stages (p < 0.05). **c** Temporal trends of the top 10 hormones with the most significant changes (p < 0.05), including abscisic acid (ABA), salicylic acid (SA), 2-Methylthio-cis-zeatin riboside (2MeScZR), indole-3-acetic acid (IAA), and trans-zeatin (tZ). The blue dots represent single seeds, and the red dots represent double seeds. The value of each point represents the mean hormone concentration (n = 3; **±** SD)

To investigate the hormonal regulation of seed germination, we performed absolute quantification of hormones in *E. saxatilis* seeds at five time points (24 h, 48 h, 72 h, 96 h, and 120 h). Seed morphology at these stages is shown in Fig. 6A. Standard curves and nine major hormone classes for these hormones are provided in Table 2 S. In terms of hormone fluctuations during germination process, the content of double seeds decreased from (34872.03 ± 5283.69) ng/mL to (23555.47 ± 2195.42) ng/mL, a decrease of 32.45%. The content of a single seed decreased from (44306.25 ± 3758.71) ng/mL to (21847.35 ± 1849.83) ng/mL, a decrease of 50.69%. By comparing the experimental data with the hormone database, we detected and quantified 66 hormones (Table 3 S), of which 45 showed significant differences (Fig. 6b). The top 10 hormones with the most significant changes included abscisic acid (ABA), 2-Methylthio-cis-zeatin riboside (2MeScZR), indole-3-acetyl-L-aspartic acid (IAA-ASP), trans-zeatin (tZ), salicylic acid (SA) and so on.

ABA and SA: Both hormones accumulated gradually over time, reaching their peak levels in the shed seed coat at 120 h. 2MeScZR, IAA, IAA-ASP, IPR, and t-CA: These hormones showed a decreasing trend over time. Single vs. Double Seed Hormone Dynamics: The fluctuation of hormone content in single seeds was significantly higher than in double seeds (Fig. 6c). Single seeds exhibited faster increases and more pronounced decreases in hormone levels (*p* < 0.05), suggesting greater metabolic activity and hormonal regulation compared to double seeds. This dynamic hormonal profile may contribute to the reproductive advantage of single-seed variants in high-altitude environments.

### Transcriptome and hormone co-expression analysis

Seed germination is a highly complex biological process regulated by dynamic changes in multiple plant hormones. Our study revealed that this process involves hormones such as ABA, JA, IPR, tZ, and GA7. To further elucidate the transition from seed dormancy to germination, we analyzed differentially expressed genes (DEGs) and differentially accumulated metabolites (DAMs) across different stages and visualized their interactions through a co-expression network.

**Fig. 7:**
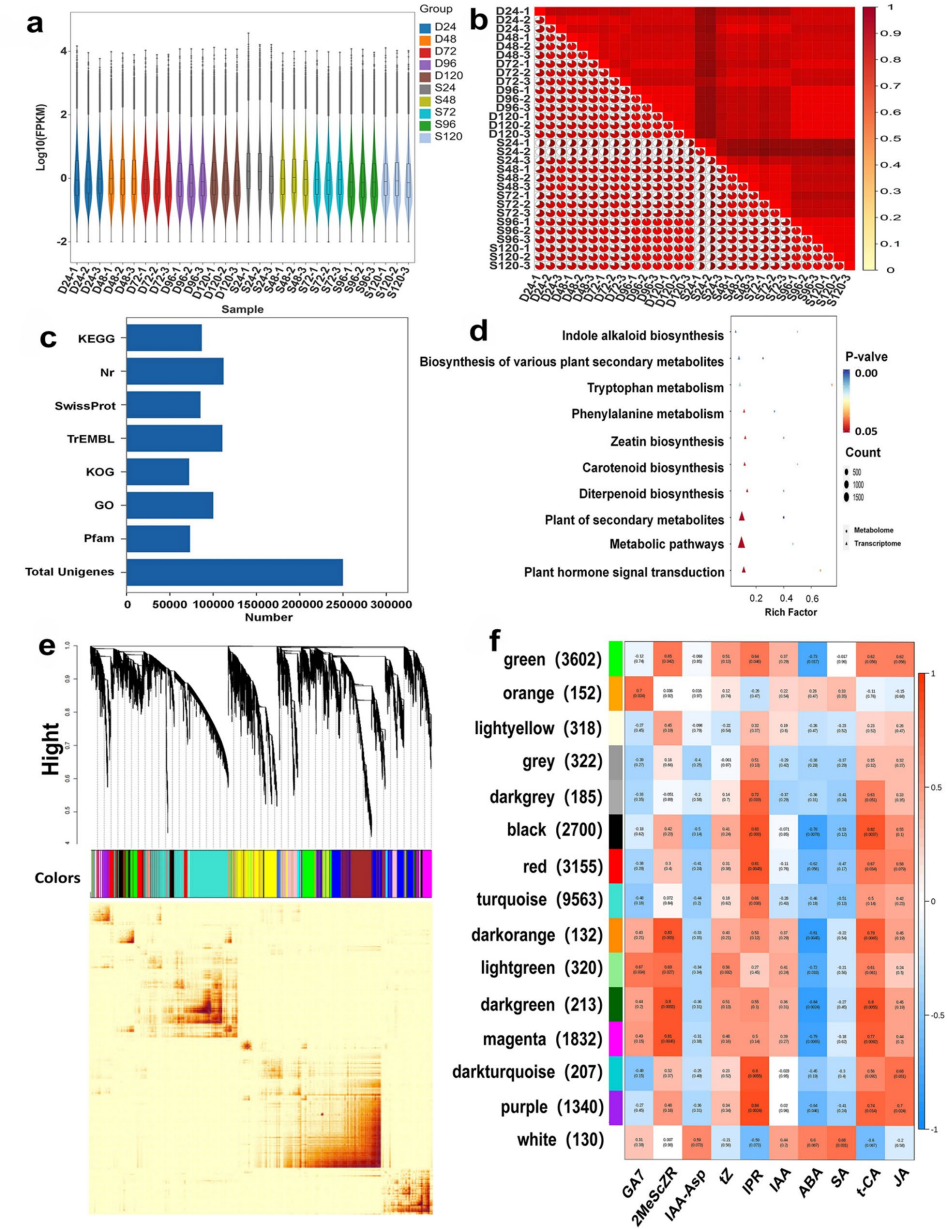
Transcriptome and hormone co-expression analysis during seed germination in *E. saxatilis. ***a** Violin plot of overall gene expression levels across samples. The size of the violin represents the distribution of gene expression levels, with single seeds showing higher expression than double seeds. **b** Heatmap of sample correlation based on the Pearson correlation coefficient (r). **c** Bar plot showing the number of gene annotations in different databases. **d** Bubble plot of the top 10 enriched pathways from transcriptome and hormone co-expression analysis. The size of the bubbles represents the number of co-expressed differential metabolites, and the color indicates the significance of enrichment (p < 0.05). **e** Hierarchical cluster tree showing co-expression modules identified by weighted gene co-expression network analysis (WGCNA). **f** Module-hormone association analysis. The heatmap shows the correlation between gene modules and hormone levels, highlighting significant associations

The results revealed that the expression level of genes in single seeds was significantly higher than in double seeds (Fig. 7a), The number of FPKM in S is 1.5 times that of D. The Pearson correlation coefficient (r) was used to evaluate the correlation between biological replicates (Fig. 7b). A total of 249,948 genes were annotated by comparing the Unigene sequences with multiple databases (Fig. 7c). Comparative analysis between single and double seeds identified 116,579 differentially expressed genes (DEGs), including 37,461 up-regulated and 29,368 down-regulated genes. Pathway enrichment analysis of these DEGs revealed the top 10 enriched pathways (Fig. 7d). To explore the dynamic transition from dormancy to germination, weighted gene co-expression network analysis (WGCNA) was performed. A total of 29 modules were identified, each represented by a distinct color (Fig. 7e). The co-expression network analysis revealed strong correlations between hormone levels and gene expression (Fig. 7f).

**Fig. 8:**
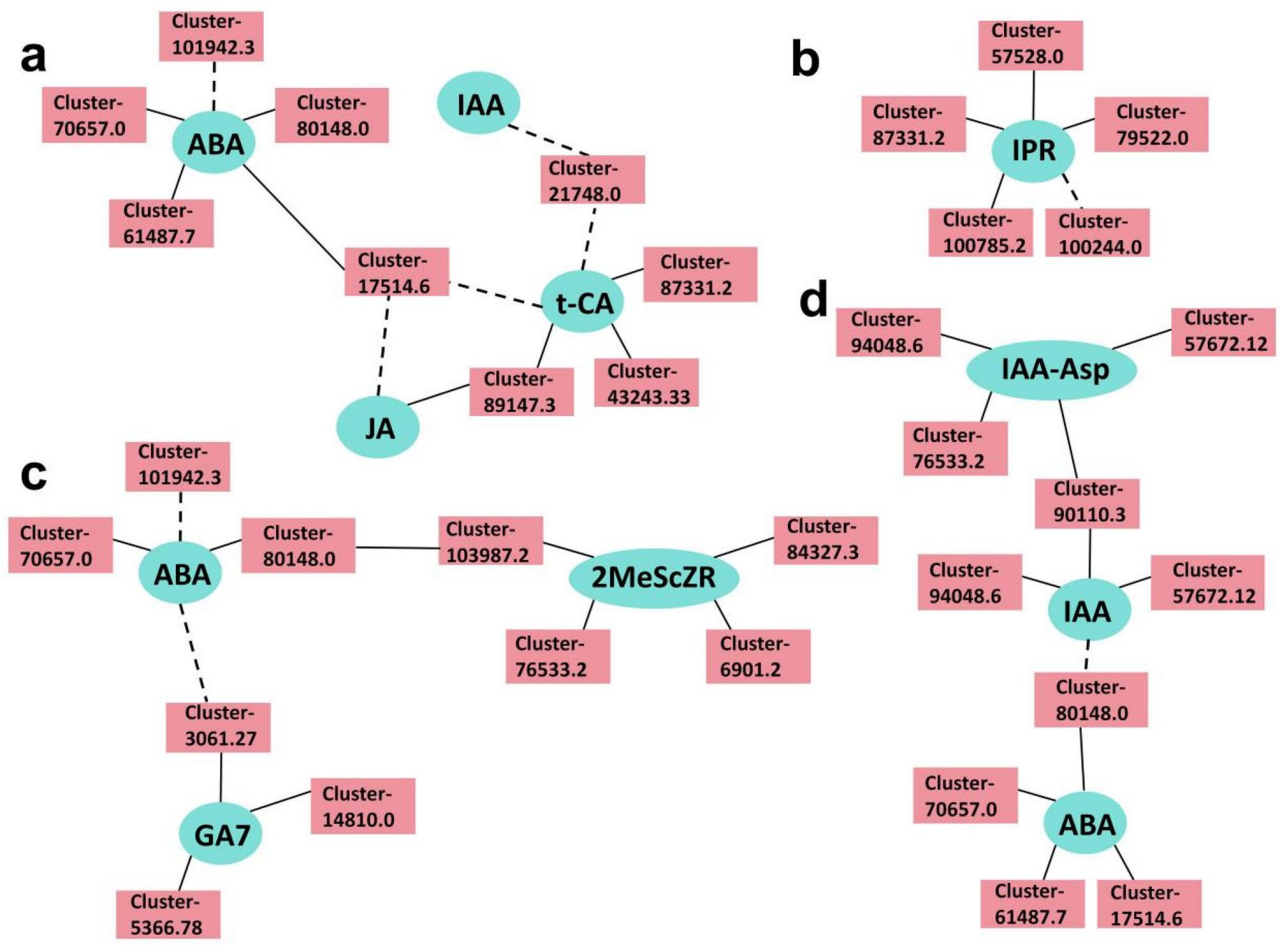
Co-expression network analysis of differentially expressed metabolites (DEMs) and differentially expressed genes (DEGs) based on Pearson correlation. **a** Interaction network between 4 DEMs and 9 DEGs in the KEGG pathway “Metabolic pathways” (ko01100). **b** Interaction network of N6-isopentenyladenosine (IPR)-associated genes. No common interacting genes were identified, suggesting a unique regulatory mechanism for IPR. **c** Interaction network between 3 DEMs and 10 DEGs in the KEGG pathway “Biosynthesis of secondary metabolites” (ko01110). **d** Interaction network of 10 DEGs and 3 DEMs in the KEGG pathway “Plant hormone signal transduction” (ko04075). Note: In the networks, pink edges represent DEMs, and blue edges represent DEGs. Solid lines indicate positive correlations (*r* > 0.8, *p* < 0.05), while dotted lines indicate negative correlations (*r* < −0.8, *p* < 0.05). The length of the line segments does not carry any significance

Specifically, changes in ABA were correlated with the expression of 2MeScZR (CK), SA, IAA (Auxin), GA7 (GAs), IPR (CK), and t-CA (SA). The co-expression network (*r* > 0.80, *p* < 0.05) highlights significant correlations between genes and metabolites (Fig. 8). Hormone-associated genes were screened and mapped to three major KEGG pathways. Metabolic pathways (ko01100): this pathway encompasses a broad range of metabolic activities essential for seed germination (Fig. 8a). Biosynthesis of secondary metabolites (ko01110): genes in this pathway are involved in the production of secondary metabolites that may play roles in stress response and adaptation (Fig. 8c). Plant hormone signal transduction (ko04075): this pathway includes genes directly involved in hormone signaling and regulation, particularly ABA synthesis and response (Fig. 8d). Further analysis of hormone-specific gene networks revealed that genes associated with ABA biosynthesis were closely linked to metabolic pathways, while no common interacting genes were identified for IPR (Fig. 8b). This suggests that ABA plays a central role in coordinating metabolic and hormonal changes during germination, whereas IPR may function through distinct regulatory mechanisms.

## Discussion

### Ecological challenges and plant conservation in Xizang

The unique geographical and climatic conditions of Xizang present significant ecological challenges. Most regions in Xizang face severe drought and water scarcity [34, 35], primarily due to the fact that a substantial portion (8,850 cubic kilometers, which translates to about 8 trillion cubic meters of water) of its water resources is stored in glacial form. The high-altitude environment, coupled with historical rain erosion, has led to widespread soil degradation, making ecological conservation a critical and long-term priority for the region. The ecological environment in Xizang is both fragile and highly sensitive to environmental changes. Exacerbated by climate change, which has driven the low vegetation coverage, migration or extinction of numerous species unable to adapt to the harsh conditions [36–38]. These factors underscore the importance of plant conservation efforts in Xizang, not only for maintaining biodiversity but also for ensuring the long-term survival of plant species in this ecologically vulnerable region.

The genus *Ephedra* is the largest and most diverse group within the Ephedraceae family, with species distributed across a wide range of global habitats. Different *Ephedra* species exhibit remarkable adaptability to varying climatic conditions, although the majority thrive in arid and semi-arid regions, where they demonstrate exceptional resilience to harsh environmental conditions such as extreme temperatures, low water availability, and poor soil quality [9, 35, 36]. For instance, *E. sinica* is predominantly found in desert plains and has been extensively studied and cultivated for its role in soil stabilization and desert greening [9, 35, 36]. Similarly, *E. equisetina*, another dominant species in plain areas, is valued for its rapid growth rate and high medicinal content, making it a reliable source for large-scale medicinal cultivation [6, 39]. To screen for *Ephedra* that can grow rapidly and provide stable medicinal sources, it is necessary to start studying *Ephedra* species and select *Ephedra* seeds with high vitality in high-altitude areas. Based on the statistical surveys of *Ephedra* species in Xizang conducted by Qin [11] and Yu [10] et al., this study provides a preliminary assessment of *Ephedra* resource distribution in the region. The findings indicate that *Ephedra* species are primarily concentrated in southern Xizang, with six species identified. Among these, *E. saxatilis* is the most abundant (Fig. 1). Seeds from six high-altitude *Ephedra* species were collected for analysis (Fig. 2a). Phenotypic observations revealed variations in seed morphology, particularly in the number of seeds per bract.

Germination rate and physiological indicators were measured for both single and double seeds. The results showed that *E. saxatilis*, *E. intermedia*, and *E. monosperma* exhibited significantly higher germination rates compared to the other three species, with single seeds demonstrating superior germination rates over double seeds (Fig. 2j). Scanning electron microscopy (SEM) analysis further revealed that the number of surface burrs and folds on seeds positively correlated with germination rates (Fig. 2g). This phenomenon may be attributed to the increased contact area between single seeds and water, facilitating sufficient seed swelling and enhancing germination efficiency.

### Metabolic dynamic response to high germination rate of seeds

The seed germination rate of *E. saxatilis* is the highest compared to the other *Ephedra* species. A higher germination rate indicates greater vitality at high altitudes, In our study, seeds that germinate under optimal laboratory conditions would be likely to survive in extreme high-altitude field conditions (e.g., low temperature, severe drought and high UV exposure). The significantly higher germination rate of *E. saxatilis* single-seed variants (*p* < 0.05) suggests stronger inherent vitality (Fig. 2j), which may translate into a competitive advantage in harsh environments. In order to reveal whether this high germination rate is determined by the metabolic rate, so as to better reveal the selection of the adaptability of *E. saxatilis* seeds under harsh conditions. We conducted metabolomics methods. Our results revealed that *E. saxatilis* displayed a significantly faster overall metabolic rate compared to other species. As germination progressed (24 h, 48 h, 72 h), metabolites in all three species showed progressive accumulation (Figs. 3e and 4b). The pathway classification of these metabolites shows obvious temporal dynamics: the metabolites of phenylpropanoid, flavonoid, and secondary metabolism pathways increase significantly, while those of carbohydrate and amino acid pathways gradually decrease (Fig. 4h). This indicates that *Ephedra* seeds give priority to the biosynthesis of flavonoids and hormones during germination rather than energy storage. Energy storage molecules may provide raw materials for the synthesis of flavonoids and hormones for the germination of *Ephedra* seeds.

The observed metabolic changes are consistent with the hypothesis that high-altitude adaptation requires efficient resource allocation. Specifically, *E. saxatilis* single seeds outperform two-seed seeds and other species in metabolic efficiency (Fig. 5e-h), which might be because it can utilize amino acids and carbohydrates more effectively to generate energy and cope with stress [40]. Similarly, Vincent et al. [41] identified dynamic shifts in phenylpropanoid and flavonoid metabolites during *Carthamus tinctorius* L. seed development, underscoring the importance of these pathways in stress adaptation. Notably, our study extends these principles to high-altitude species, revealing that *E. saxatilis* uniquely optimizes metabolic flux toward stress tolerance mechanisms.

### Hormonal dynamics in seed germination

Through metabolomics, we discovered that the changes in hormones were highly significant during the seed germination process. Therefore, we chose to conduct an absolute quantitative analysis of hormone substances. Plant hormones are critical signaling molecules that regulate growth, development, and stress responses, acting as promoters or inhibitors of plant physiological processes [42–45]. The higher the hormone content, the higher the seed vitality may be. In this study, through absolute quantitative analysis of hormone levels during seed germination, it was found that among 66 hormones, 45 showed statistically significant differences. During the germination process, the hormone content of double seeds decreased from (34872.03 ± 5283.69) ng/mL to (23555.47 ± 2195.42) ng/mL, a decrease of 32.45%. The content of a single seed decreased from (44306.25 ± 3758.71) ng/mL to (21847.35 ± 1849.83) ng/mL, a decrease of 50.69%. The top ten differentially expressed hormones, including 2MeScZR, IAA, IAA-ASP, IPR and t-CA, showed a downward trend over time. Hormone fluctuations in single seeds were faster than those in double seeds (Fig. 6a-c). These results suggest that single seeds may have hormonal advantages, which could be one of the reasons for their high germination rate. The overall hormone content in single seeds is also higher than that in double seeds, which is consistent with the hypothesis in this study that a higher hormone content may be associated with a higher seed germination rate.

Consistent with previous studies, gibberellins (GAs) play a pivotal role in seed germination, root and shoot elongation, and flowering, with their levels directly influencing germination rates [43]. Additionally, abscisic acid (ABA) is a key regulator of stress responses and developmental processes such as stomatal closure, leaf senescence, and seed dormancy [44–46]. Nguyen et al. [46] demonstrated that ABA enhances plant stress tolerance, while Banerjee et al. [47] and Ali et al. [48] highlighted the antagonistic relationship between ABA and melatonin in regulating stress responses. These results align with our observations, where ABA accumulation likely regulates seed coat shedding and seedling maturation (Fig. 6a).

### Transcriptome and hormones analysis of key substances and genes

After determining that the single seed of *E. saxatilis* is the dominant seeds under high altitudes based on germination rate, metabolic rate and hormone changes, we conducted a combined transcriptome and hormone analysis on the germinated seeds to identify key genes regulating hormones. This further clarified the mechanism and coping strategies of seed germination under high-altitude conditions. Transcriptomic analysis revealed significant gene expression changes during the transition from dormancy to germination, particularly in pathways related to metabolic processes, secondary metabolite biosynthesis, and plant hormone signal transduction (Fig. 7d). Weighted gene co-expression network analysis (WGCNA) and co-expression network analysis further highlighted the antagonistic relationship between ABA and other hormones, including 2MeScZR (CK), tZ (CK), SA, IAA, GA7, JA, and t-CA (Fig. 7f). These findings underscore the critical role of hormonal balance in seed germination, with ABA acting as an inhibitor and GAs/CKs promoting cell proliferation and dormancy release [49–52].

The multi-omics method is an effective joint analysis method. Combined with the database, it can quickly screen for differential DEMs and DEGs in plants and correlation analysis to identify key differential metabolites and analyze the dynamic changes of plants. Zhao et al. [25] used LC-MS to analyze the temporal metabolomics of cashew fruit and apples, and identified the accumulation of specific metabolites. They found that phosphatidylinositol is the main component of unsaturated glycerophospholipids, and identified a transcription factor through transcriptomics, which is a potential synthetic factor for phosphatidylinositol, explaining the partial metabolic network of cashews during development. Ning et al. [24] conducted transcriptome and metabolome study on *Sinopodophyllum hexandrum* by screening different treatment layers. When studying *S. hexandrum* for different treatment times, they found that the activity of *S. hexandrum* reached its highest at 120 days and identified antagonistic and synergistic trends of related metabolites, gene network of hormone was constructed. In this study, through metabolomics, absolute quantification of hormones and transcriptomics, *Ephedra* single seeds were selected as the dominant seeds at high altitudes. In adapting to high-pressure environments, plants may produce single seeds with higher hormone content for their own survival. However, the specific reasons for the emergence of single seeds will be our subsequent research.

### Single seed phenotype and ecological implications in high attitude

The observed single-seed phenotype could result from either environmentally induced plasticity. Arshad et al. [53]. reported single-seed phenotypes in *Aethionema arabicum* and suggested that this trait arose may play a key role in regulating survival strategies under the challenges of global climate change. In high-altitude areas, climate warming, early melting of snow and changes in precipitation could affect the number of seeds in the embryo, germination and the emergence of seedling [54]– [55]. Noor et al. [56] indicated that the low seeds of rice SR4 in high-altitude areas is due to abiotic stresses, such as cold stress and other phenomena. Conversely, a higher seed setting rate of *Brassica napus. L.* was found which is due to higher sunlight at high altitudes [57].

In most *Ephedra* species, cones typically contain two seeds. However, this study observed variations in seed number per bract, including single and triple seeds, with *E. monosperma* being an exception (single seeds are normal, while double seeds are dominant variants). However, whether this trait is stably inherited within the species requires further long-term investigation.The formation of single seeds may result from both genetic and environmental factors [58].We hypothesize that single-seed formation could be an evolutionary strategy to enhance survival under harsh conditions and need to be further deep research on evolutionary strategy.

## Conclusion

This study comprehensively characterized the physiological and molecular adaptations of high-altitude *Ephedra* seeds through multi-omics approaches. By screening germination rates and seed weight, *E. saxatilis*, *E. intermedia*, and *E. monosperma* were identified as high-performance species, with *E. saxatilis* exhibiting the fastest metabolic rate and emerging as the dominant species among the three. Temporal metabolomics analysis revealed that *E. saxatilis* optimizes metabolic flux toward stress-responsive pathways, such as trans-Cinnamic acid and Hippuric Acid in phenylpropanoid pathway were increased. Myricetin, quercetin, apigenin amd syringetin in flavonoid biosynthesis pathway were increased, while efficiently utilizing energy reserves during germination. Absolute quantification of hormones further demonstrated that single seeds exhibit more dynamic hormonal changes (decreased 50.69%) compared to normal seeds, highlighting their superior adaptability. Co-expression network analysis identified key hormone-gene interactions, particularly the antagonistic relationship between ABA and 2MeScZR, SA, IAA, GA7, IPR, t-CA, which play a decisive role in seed germination and dormancy release. These findings position single seeds as the dominant variants in high-altitude environments, offering a promising target for future research. Subsequent studies will focus on elucidating the molecular mechanisms underlying single seed formation, with the aim of enhancing seed viability and ensuring the ecological continuity of high-altitude *Ephedra* species.

## Supplementary Information

Supplementary Material 1.

Supplementary Material 2.

Supplementary Material 3. Tables S1 to S3, hormone dataset, transcriptome dataset, metabolic dataset.

Supplementary Material 4.

## Data Availability

The data in the manuscript have all been uploaded to the system. The RNA sequencing data associated with transcription profiles in this study have been deposited in NCBI database with accession number PRJNA1238763 (https://www.ncbi.nlm.nih.gov/bioproject/PRJNA1238763).
