# Supplementary material for: Multi-omics profiling reveals single-seed mutants of Ephedra saxatilis as dominant variants in high-altitude Xizang
Source: BMC Plant Biol. 2025 Aug 22;25:1118. doi: 10.1186/s12870-025-07153-x (PMC12372293; doi:10.1186/s12870-025-07153-x)
Supplement: Supplementary file 1 — Supplementary Material 1. [file 12870_2025_7153_MOESM1_ESM.docx]

**Table S1 Collected information of six species of Ephedra (longitude, latitude, altitude, species)**

| **Number** | **Sample site** | **Altitude/m** | **Longitude** | **Latitude** | **Species** |
| --- | --- | --- | --- | --- | --- |
| **1** | Jilong | 4207 | 28.718923 | 85.275637 | ***E. saxatilis*** |
| **2** | Nilamu | 4164 | 28.254511 | 86.010916 |  |
| **3** | Dingri | 4107 | 28.489123 | 87.563501 |  |
| **4** | Pali | 4386 | 27.763071 | 89.168505 |  |
| **5** | Lhasa | 4060 | 29.690746 | 91.107313 |  |
| **6** | Lhasa | 4761 | 29.726199 | 91.124900 |  |
| **7** | Lhasa | 4217 | 29.452461 | 91.956135 |  |
| **8** | Lhasa | 3707 | 29.680833 | 91.084444 |  |
| **9** | Lhasa | 3862 | 29.697037 | 91.133238 |  |
| **10** | Linzhou | 4143 | 30.176107 | 91.279017 |  |
| **11** | Linzhou | 4431 | 30.137620 | 91.245603 |  |
| **12** | Linzhou | 4213 | 30.077168 | 91.277724 |  |
| **13** | Linzhou | 4738 | 30.110978 | 91.253921 |  |
| **14** | Linzhou | 3810 | 29.882903 | 91.184652 |  |
| **15** | Linzhou | 4150 | 30.099718 | 91.280816 |  |
| **16** | Mozhu | 4104 | 29.894347 | 91.958786 |  |
| **17** | Mozhu | 3975 | 29.976797 | 91.921600 |  |
| **18** | Mozhu | 4831 | 29.795829 | 92.361708 |  |
| **19** | Gongbu | 3245 | 29.871901 | 93.662516 |  |
| **20** | Gongbu | 3317 | 29.958307 | 93.658542 |  |
| **21** | Peigu | 4864 | 28.932094 | 85.405531 | ***E. gerardiana*** |
| **22** | Langkazi | 4650 | 28.896348 | 90.291065 |  |
| **23** | Jiangzi | 4121 | 28.867456 | 89.727894 |  |
| **24** | Nielamu | 4853 | 28.584822 | 86.143373 |  |
| **25** | Longzi | 3515 | 28.383116 | 92.732111 |  |
| **26** | Jiali | 4283 | 30.4324 | 91.9411 |  |
| **27** | Chayu | 4686 | 29.328914 | 97.040489 | ***E. minuta*** |
| **28** | Chayu | 3346 | 29.942513 | 97.426159 |  |
| **29** | Chayu | 4197 | 29.329320 | 97.103368 |  |
| **30** | Chayu | 4613 | 29.319152 | 96.968487 | ***E. likiangensis*** |
| **31** | Chayu | 3400 | 29.151668 | 97.204653 |  |
| **32** | Chayu | 4153 | 29.334495 | 97.064212 |  |
| **33** | Chayu | 3415 | 29.161662 | 97.204619 | ***E. intermedia*** |
| **34** | Chayu | 2768 | 28.942518 | 97.426159 |  |
| **35** | Jiacha | 3179 | 29.116908 | 92.692290 |  |
| **36** | Milin | 2970 | 29.117893 | 93.815724 |  |
| **37** | Basu | 4101 | 30.278912 | 97.270229 | ***E. monosperma*** |
| **38** | Cuona | 4317 | 27.979415 | 91.926124 |  |
| **39** | Longzi | 4218 | 28.160222 | 91.961947 |  |

**Table S2 109 hormones name, class, abbreviation, standard curves**

| **Name (Abbreviation)** | **Class** | **Standard curve** | **r^2^** |
| --- | --- | --- | --- |
| ABA-glucosyl ester （ABA-GE） | ABA | y = 0.02753 x + 8.531944 | 0.99131 |
| Abscisic acid（ABA） | ABA | y = 0.06980 x - 0.00161 | 0.99795 |
| Abscisic aldehyde（ABA-ald） | ABA | y = 0.00984 x + 0.00164 | 0.99073 |
| L-tryptophan（TRP） | Auxin | y = 0.00569 x + 0.00686 | 0.9998 |
| Tryptamine（TRA） | Auxin | y = 0.14320 x + 6.877434 | 0.99977 |
| 2-oxindol3-acetic acid（OxIAA） | Auxin | y = 0.01146 x + 3.244485 | 0.99119 |
| Indol3-acetyl-L-aspartic acid（AA-Asp） | Auxin | y = 0.02035 x + 2.415134 | 0.99073 |
| 1-O-indol-3-ylacetylglucose（IAA-Glc） | Auxin | y = 8.710944 x + 4.226314 | 0.99037 |
| 3-Indole acetamide（IAM） | Auxin | y = 0.36662 x + 0.00556 | 0.99774 |
| Indol3-acetyl glutamic acid（IAA-Glu） | Auxin | y = 0.13241 x - 4.845044 | 0.99176 |
| Indol3-acetyl glycine（IAA-Gly） | Auxin | y = 0.34508 x - 2.065495 | 0.99809 |
| Indol3-lactic acid（ILA） | Auxin | y = 0.01437 x - 0.00578 | 0.99412 |
| N-(3-Indolylacetyl)-L-alanine（IAA-Ala） | Auxin | y = 0.21274 x - 5.465694 | 0.99908 |
| Indol3-carboxylic acid（ICA） | Auxin | y = 0.00728 x + 2.286084 | 0.99179 |
| Indol3-carboxaldehyde（ICAld） | Auxin | y = 0.04651 x + 0.00283 | 0.99158 |
| Indol3-acetic acid（IAA） | Auxin | y = 0.01153 x + 2.982514 | 0.99686 |
| 3-Indoleacrylic acid（IA） | Auxin | y = 0.01456 x - 1.704714 | 0.99174 |
| N-(3-Indolylacetyl)-L-valine（IAA-Val） | Auxin | y = 0.81572 x - 0.00298 | 0.99062 |
| 3-Indolepropionic acid（IPA） | Auxin | y = 0.04182 x + 1.186214 | 0.9947 |
| Indol3-acetyl-L-glutamic acid dimethyl ester（IAA-Glu-diMe） | Auxin | y = 0.05503 x - 0.00782 | 0.99009 |
| Indol3-acetyl-L-tryptophan（IAA-Trp） | Auxin | y = 0.34235 x - 0.00604 | 0.99166 |
| N-(3-Indolylacetyl)-L-leucine（IAA-Leu） | Auxin | y = 0.96705 x + 0.00755 | 0.99896 |
| Indol3-butyric acid（IBA） | Auxin | y = 0.02284 x + 0.07594 | 0.99577 |
| 3-Indoleacetonitrile（IAN） | Auxin | y = 0.02640 x + 0.00137 | 0.99034 |
| N-(3-Indolylacetyl)-L-phenylalanine（IAA-Phe） | Auxin | y = 0.64102 x - 0.00100 | 0.99198 |
| Methyl indol3-acetate（MEIAA） | Auxin | y = 0.08391 x + 5.584824 | 0.99023 |
| Indol3-acetyl-L-valine methyl ester（IAA-Val-Me） | Auxin | y = 1.71031 x + 0.03518 | 0.99606 |
| Indole（Indole） | Auxin | y = 7.314215 x + 1.322244 | 0.99067 |
| Indol3-acetyl-L-leucine methyl ester（IAA-Leu-Me） | Auxin | y = 2.22821 x + 0.00246 | 0.9916 |
| Indol3-acetyl-L-phenylalanne methyle ester（IAA-PhMe） | Auxin | y = 1.44908 x + 0.00139 | 0.99278 |
| 9-Ribosyl-trans-zeatin 5'-monophosphate（tZRMP） | CK | y = 0.01123 x + 3.338754 | 0.99008 |
| trans-Zeatin-O-glucoside（tZOG） | CK | y = 0.08556 x + 2.969424 | 0.99915 |
| cis-Zeatin riboside monophosphate（cZRMP） | CK | y = 0.01927 x + 6.614954 | 0.9902 |
| trans-Zeatin（tZ） | CK | y = 0.13848 x + 9.767284 | 0.99533 |
| Dihydrozeatin（DZ） | CK | y = 0.04392 x + 9.660414 | 0.99684 |
| trans-Zeatin-9-glucoside（tZ9G） | CK | y = 0.08226 x + 5.260264 | 0.99303 |
| Dihydrozeatin-7-glucoside（DHZ7G） | CK | y = 0.34456 x + 0.00131 | 0.99047 |
| cis-Zeatin（cZ） | CK | y = 0.07244 x + 9.454164 | 0.99703 |
| cis-Zeatin-9-glucoside（cZ9G） | CK | y = 0.45282 x + 8.923295 | 0.99469 |
| Dihydrozeatin-O-glucoside riboside（DHZROG） | CK | y = 0.30272 x + 0.00187 | 0.99869 |
| cis-Zeatin-O-glucoside riboside（cZROG） | CK | y = 0.03909 x + 6.253316 | 0.99247 |
| para-Topolin（pT） | CK | y = 0.07950 x + 2.828654 | 0.99119 |
| 4-[[(9-beta-D-Glucopyranosyl-9H-purin-6-yl)amino]methyl]phenol（pT9G） | CK | y = 0.16482 x + 0.00489 | 0.99305 |
| trans-Zeatin riboside（tZR） | CK | y = 0.13499 x + 0.00137 | 0.99031 |
| Dihydrozeatin ribonucleoside（DHZR） | CK | y = 0.09610 x + 3.687334 | 0.99084 |
| cis-Zeatin riboside（cZR） | CK | y = 0.13011 x + 8.523124 | 0.99066 |
| N6-Isopentenyl-adenin7-glucoside（iP7G） | CK | y = 0.33547 x + 6.428104 | 0.99564 |
| meta-Topolin（mT） | CK | y = 0.11232 x + 6.486344 | 0.99187 |
| meta-Topolin-9-glucoside（mT9G） | CK | y = 0.18720 x + 7.240595 | 0.99201 |
| N6-Benzyladenin7-glucoside（BAP7G） | CK | y = 0.46002 x + 3.639844 | 0.99217 |
| N-6-iso-pentenyladenosin5'-monophosphate（iPRMP） | CK | y = 0.04479 x + 0.00123 | 0.99174 |
| Kinetin（K） | CK | y = 0.08362 x + 6.689754 | 0.99108 |
| Kinetin-9-glucoside（K9G） | CK | y = 0.24168 x + 0.00126 | 0.99005 |
| para-Topolin riboside（pTR） | CK | y = 0.18749 x + 5.863154 | 0.99274 |
| meta-Topolin riboside（mTR） | CK | y = 0.23044 x + 0.00455 | 0.99256 |
| ortho-Topolin-9-glucoside（oT9G） | CK | y = 0.11142 x + 2.514337 | 0.99224 |
| N6-Isopentenyl-adenin9-glucoside（iP9G） | CK | y = 0.20534 x + 2.472354 | 0.99049 |
| N6-isopentenyladenine（IP） | CK | y = 0.05596 x + 7.335904 | 0.99323 |
| ortho-Topolin（oT） | CK | y = 0.14315 x + 1.212154 | 0.9915 |
| Kinetin riboside（KR） | CK | y = 0.28030 x + 1.642264 | 0.99118 |
| N6-Benzyladenin9-glucoside（BAP9G） | CK | y = 0.37687 x + 5.066544 | 0.99432 |
| 6-Benzyladenine（BAP） | CK | y = 0.15117 x + 0.00124 | 0.99629 |
| 2-Chloro-trans-zeatin（2CltZ） | CK | y = 0.09747 x + 2.609484 | 0.99815 |
| ortho-Topolin riboside（oTR） | CK | y = 0.16695 x - 2.272424 | 0.9901 |
| 2-Methylthio-cis-zeatin（2MeScZ） | CK | y = 0.02120 x + 2.142225 | 0.99766 |
| N6-isopentenyladenosine（IPR） | CK | y = 0.05842 x + 6.589994 | 0.99515 |
| 2-Methylthio-cis-zeatin riboside（2MeScZR） | CK | y = 0.12893 x + 0.00181 | 0.9966 |
| 6-Benzyladenosine（BAPR） | CK | y = 0.42811 x + 7.054394 | 0.99011 |
| 2-Methylthio-N6-isopentenyladenosine（2MeSiPR） | CK | y = 0.02604 x + 2.125754 | 0.99591 |
| 2-Methylthio-N6-isopentenyladenine（2MeSiP） | CK | y = 0.01637 x + 5.455715 | 0.99705 |
| 1-Aminocyclopropanecarboxylic acid（ACC） | ETH | y = 26290.15487 x + 27294.00492 | 0.99178 |
| Gibberellin A8（GA8） | GA | y = 0.16031 x + 2.387494 | 0.99587 |
| Gibberellin A29（GA29） | GA | y = 0.00655 x + 6.656324 | 0.9917 |
| Gibberellin A3（GA3） | GA | y = 0.14283 x + 0.00214 | 0.99385 |
| Gibberellin A1（GA1） | GA | y = 0.03700 x - 0.01373 | 0.99317 |
| Gibberellin A6（GA6） | GA | y = 0.14851 x + 7.470474 | 0.99569 |
| Gibberellin A19（GA19） | GA | y = 0.02861 x + 0.00717 | 0.99805 |
| Gibberellin A5（GA5） | GA | y = 0.04718 x + 0.00134 | 0.9901 |
| Gibberellin A20（GA20） | GA | y = 0.01497 x + 0.00268 | 0.99778 |
| Gibberellin A44（GA44） | GA | y = 0.03347 x + 8.310774 | 0.99133 |
| Gibberellin A34（GA34） | GA | y = 0.17782 x + 9.497374 | 0.99064 |
| Gibberellin A51（GA51） | GA | y = 0.02073 x + 0.00399 | 0.99052 |
| Gibberellin A53（GA53） | GA | y = 0.04773 x + 0.01735 | 0.9926 |
| Gibberellin A7（GA7） | GA | y = 0.63563 x + 0.00753 | 0.9952 |
| Gibberellin A4（GA4） | GA | y = 0.04551 x + 5.910624 | 0.99653 |
| Gibberellin A24（GA24） | GA | y = 0.05745 x - 0.02446 | 0.99025 |
| Gibberellin A15（GA15） | GA | y = 0.03556 x + 4.947464 | 0.99194 |
| Gibberellin A9（GA9） | GA | y = 0.05179 x + 0.00336 | 0.99436 |
| Gibberellin A12 aldehyde（GA12-ald） | GA | y = 0.07553 x - 0.00225 | 0.99208 |
| 12-Hydroxyjasmonic acid（12-OH-JA） | JA | y = 0.09676 x + 0.23417 | 0.99011 |
| Jasmonat1-aminocyclopropan1-carboxylic acid（JA-ACC） | JA | y = 0.20581 x + 0.00335 | 0.9923 |
| Jasmonic acid（JA） | JA | y = 0.07643 x + 9.044254 | 0.99696 |
| N-[(-)-Jasmonoyl]-(L)-valine（JA-Val） | JA | y = 0.96107 x + 0.00267 | 0.99005 |
| Dihydrojasmonic acid（H2JA） | JA | y = 0.07857 x + 0.00352 | 0.99 |
| Jasmonoyl-L-isoleucine（JA-ILE） | JA | y = 0.08253 x + 3.633954 | 0.99017 |
| N-[(-)-Jasmonoyl]-(l)-phenalanine（JA-Phe） | JA | y = 0.50150 x + 7.564424 | 0.99118 |
| 3-oxo-2-(2-(Z)-Pentenyl) cyclopentan1-butyric acid（OPC-4） | JA | y = 0.08010 x + 0.02318 | 0.99486 |
| Methyl jasmonate（MEJA） | JA | y = 0.38491 x - 0.00567 | 0.99896 |
| 3-oxo-2-(2-(Z)-Pentenyl)cyclopentan1-hexanoic acid（OPC-6） | JA | y = 0.10184 x + 0.04886 | 0.99073 |
| cis(+)-12-Oxophytodienoic acid（OPDA） | JA | y = 2.00324 x + 0.02652 | 0.99227 |
| Melatonine（MLT） | MLT | y = 1.24510e6 x + 1120.42358 | 0.99322 |
| L-Phenylalanine（Phe） | SA | y = 1234.24233 x - 621.81818 | 0.99036 |
| Salicylic acid 2-O-β-glucoside（SAG） | SA | y = 0.06579 x + 0.00244 | 0.99455 |
| 2-Methoxycarbonylphenyl beta-D-glucopyranoside（MeSAG） | SA | y = 2322.39536 x + 9.76933 | 0.99128 |
| 2-Coumarate（2-Coumarate） | SA | y = 4.35765 x - 1603.10233 | 0.99007 |
| Salicylic acid（SA） | SA | y = 0.04328 x + 0.01068 | 0.99209 |
| trans-Cinnamic acid（t-CA） | SA | y = 277.25384 x - 2516.45292 | 0.99177 |
| （±）Strigol（ST） | SL | y = 1053.60105 x + 191.84905 | 0.99417 |
| 5-Deoxystrigol（5DS） | SL | y = 4.52011 x - 3.66631 | 0.9984 |

**Table S3 The absolute content of 66 hormones detected in *Ephedra***

| **Name** | **Double** | **Mean±SD** | **Single** | **Mean±SD** |
| --- | --- | --- | --- | --- |
| 2-Coumarate | D120 | 13.21±2.68 | S120 | 15.45±13.60 |
| 2-Coumarate | D96 | 30.96±19.98 | S96 | 32.82±1.81 |
| 2-Coumarate | D72 | 24.42±11.27 | S72 | 46.30±6.97 |
| 2-Coumarate | D48 | 55.80±29.62 | S48 | 44.50±9.65 |
| 2-Coumarate | D24 | 46.69±37.02 | S24 | 49.00±55.27 |
| 2MeScZ | D120 | 0.00±0.00 | S120 | 0.46±0.54 |
| 2MeScZ | D96 | 0.28±0.29 | S96 | 0.17±0.29 |
| 2MeScZ | D72 | 0.32±0.56 | S72 | 0.64±0.16 |
| 2MeScZ | D48 | 0.14±0.25 | S48 | 0.55±0.48 |
| 2MeScZ | D24 | 0.25±0.44 | S24 | 0.00±0.00 |
| 2MeScZR | D120 | 0.35±0.05 | S120 | 0.79±0.40 |
| 2MeScZR | D96 | 0.89±0.51 | S96 | 0.94±0.11 |
| 2MeScZR | D72 | 1.01±0.64 | S72 | 1.81±0.43 |
| 2MeScZR | D48 | 1.71±0.17 | S48 | 2.53±0.44 |
| 2MeScZR | D24 | 3.74±1.27 | S24 | 4.36±1.48 |
| 2MeSiP | D120 | 0.42±0.08 | S120 | 4.46±5.90 |
| 2MeSiP | D96 | 1.10±0.72 | S96 | 0.84±0.25 |
| 2MeSiP | D72 | 1.01±0.65 | S72 | 0.97±0.54 |
| 2MeSiP | D48 | 0.77±0.10 | S48 | 0.66±0.34 |
| 2MeSiP | D24 | 0.26±0.07 | S24 | 0.48±0.17 |
| 2MeSiPR | D120 | 1.67±0.19 | S120 | 2.47±0.95 |
| 2MeSiPR | D96 | 2.49±1.03 | S96 | 2.96±0.30 |
| 2MeSiPR | D72 | 2.05±1.81 | S72 | 3.47±0.61 |
| 2MeSiPR | D48 | 5.12±1.67 | S48 | 4.01±0.86 |
| 2MeSiPR | D24 | 4.21±2.17 | S24 | 3.23±2.95 |
| 5DS | D120 | 0.00±0.00 | S120 | 0.85±1.47 |
| 5DS | D96 | 1.61±1.39 | S96 | 1.71±1.48 |
| 5DS | D72 | 2.71±0.28 | S72 | 0.00±0.00 |
| 5DS | D48 | 2.78±0.41 | S48 | 2.56±0.05 |
| 5DS | D24 | 3.00±3.22 | S24 | 5.16±2.42 |
| ABA | D120 | 297.07±21.97 | S120 | 574.63±73.07 |
| ABA | D96 | 150.43±40.71 | S96 | 93.28±12.40 |
| ABA | D72 | 98.27±68.83 | S72 | 92.17±6.92 |
| ABA | D48 | 93.11±19.66 | S48 | 77.43±57.07 |
| ABA | D24 | 80.25±81.34 | S24 | 65.21±7.55 |
| ACC | D120 | 9.96±17.25 | S120 | 0.00±0.00 |
| ACC | D96 | 0.00±0.00 | S96 | 0.00±0.00 |
| ACC | D72 | 0.00±0.00 | S72 | 17.00±14.80 |
| ACC | D48 | 9.51±16.48 | S48 | 12.98±11.45 |
| ACC | D24 | 0.00±0.00 | S24 | 0.00±0.00 |
| BAP | D120 | 0.54±0.57 | S120 | 0.00±0.00 |
| BAP | D96 | 0.08±0.13 | S96 | 0.00±0.00 |
| BAP | D72 | 0.13±0.23 | S72 | 0.13±0.23 |
| BAP | D48 | 0.07±0.13 | S48 | 0.00±0.00 |
| BAP | D24 | 0.00±0.00 | S24 | 0.00±0.00 |
| BAPR | D120 | 0.05±0.08 | S120 | 0.02±0.04 |
| BAPR | D96 | 0.04±0.06 | S96 | 0.04±0.07 |
| BAPR | D72 | 0.08±0.07 | S72 | 0.06±0.10 |
| BAPR | D48 | 0.00±0.00 | S48 | 0.07±0.11 |
| BAPR | D24 | 0.00±0.00 | S24 | 0.00±0.00 |
| cZ | D120 | 0.15±0.13 | S120 | 0.27±0.32 |
| cZ | D96 | 0.15±0.26 | S96 | 0.12±0.12 |
| cZ | D72 | 0.19±0.18 | S72 | 0.00±0.00 |
| cZ | D48 | 0.11±0.19 | S48 | 0.17±0.18 |
| cZ | D24 | 2.89±4.75 | S24 | 4.40±3.83 |
| cZ9G | D120 | 0.00±0.00 | S120 | 0.75±1.30 |
| cZ9G | D96 | 0.00±0.00 | S96 | 0.00±0.00 |
| cZ9G | D72 | 0.00±0.00 | S72 | 1.55±0.10 |
| cZ9G | D48 | 1.09±1.89 | S48 | 0.00±0.00 |
| cZ9G | D24 | 0.00±0.00 | S24 | 0.00±0.00 |
| cZR | D120 | 0.00±0.00 | S120 | 0.12±0.21 |
| cZR | D96 | 0.07±0.13 | S96 | 0.00±0.00 |
| cZR | D72 | 0.00±0.00 | S72 | 0.00±0.00 |
| cZR | D48 | 0.00±0.00 | S48 | 0.00±0.00 |
| cZR | D24 | 0.00±0.00 | S24 | 0.00±0.00 |
| cZRMP | D120 | 0.00±0.00 | S120 | 0.66±1.15 |
| cZRMP | D96 | 0.00±0.00 | S96 | 0.98±1.69 |
| cZRMP | D72 | 2.80±2.62 | S72 | 2.50±2.22 |
| cZRMP | D48 | 0.00±0.00 | S48 | 1.65±2.86 |
| cZRMP | D24 | 1.99±3.44 | S24 | 0.00±0.00 |
| cZROG | D120 | 0.06±0.11 | S120 | 0.00±0.00 |
| cZROG | D96 | 0.00±0.00 | S96 | 0.03±0.06 |
| cZROG | D72 | 0.00±0.00 | S72 | 0.16±0.14 |
| cZROG | D48 | 0.27±0.25 | S48 | 0.00±0.00 |
| cZROG | D24 | 0.23±0.20 | S24 | 0.26±0.45 |
| GA12-ald | D120 | 95.77±70.15 | S120 | 7.43±3.25 |
| GA12-ald | D96 | 79.00±61.24 | S96 | 50.03±57.43 |
| GA12-ald | D72 | 27.82±14.12 | S72 | 8.18±7.43 |
| GA12-ald | D48 | 16.77±8.00 | S48 | 63.50±36.84 |
| GA12-ald | D24 | 44.45±18.50 | S24 | 37.69±25.71 |
| GA20 | D120 | 729.23±203.52 | S120 | 538.68±419.31 |
| GA20 | D96 | 813.54±56.06 | S96 | 667.85±408.95 |
| GA20 | D72 | 1315.00±956.14 | S72 | 1462.50±243.74 |
| GA20 | D48 | 981.40±539.83 | S48 | 2522.80±658.98 |
| GA20 | D24 | 1627.80±342.50 | S24 | 1142.50±210.35 |
| GA3 | D120 | 1.98±1.88 | S120 | 2.27±2.70 |
| GA3 | D96 | 1.30±1.70 | S96 | 0.71±1.22 |
| GA3 | D72 | 0.70±1.21 | S72 | 0.17±0.29 |
| GA3 | D48 | 0.00±0.00 | S48 | 0.00±0.00 |
| GA3 | D24 | 1.59±2.75 | S24 | 1.98±3.43 |
| GA5 | D120 | 6.40±5.55 | S120 | 0.00±0.00 |
| GA5 | D96 | 0.00±0.00 | S96 | 2.41±4.18 |
| GA5 | D72 | 2.57±4.45 | S72 | 5.32±4.69 |
| GA5 | D48 | 9.00±7.99 | S48 | 4.32±7.48 |
| GA5 | D24 | 4.54±7.86 | S24 | 0.00±0.00 |
| GA53 | D120 | 0.00±0.00 | S120 | 1.42±2.46 |
| GA53 | D96 | 0.00±0.00 | S96 | 0.00±0.00 |
| GA53 | D72 | 0.50±0.87 | S72 | 0.00±0.00 |
| GA53 | D48 | 0.00±0.00 | S48 | 0.90±1.56 |
| GA53 | D24 | 2.00±3.47 | S24 | 6.96±6.97 |
| GA7 | D120 | 2.32±0.72 | S120 | 0.59±0.47 |
| GA7 | D96 | 2.32±1.00 | S96 | 0.91±0.34 |
| GA7 | D72 | 1.81±0.08 | S72 | 2.23±0.34 |
| GA7 | D48 | 1.78±0.74 | S48 | 1.95±0.84 |
| GA7 | D24 | 3.25±1.22 | S24 | 1.84±1.11 |
| GA8 | D120 | 0.76±1.32 | S120 | 0.00±0.00 |
| GA8 | D96 | 0.69±1.19 | S96 | 0.00±0.00 |
| GA8 | D72 | 0.61±1.06 | S72 | 0.00±0.00 |
| GA8 | D48 | 0.00±0.00 | S48 | 2.58±0.32 |
| GA8 | D24 | 0.00±0.00 | S24 | 0.00±0.00 |
| GA9 | D120 | 0.00±0.00 | S120 | 0.85±1.47 |
| GA9 | D96 | 1.46±2.53 | S96 | 0.00±0.00 |
| GA9 | D72 | 0.69±1.20 | S72 | 0.00±0.00 |
| GA9 | D48 | 0.00±0.00 | S48 | 1.12±1.95 |
| GA9 | D24 | 18.50±32.04 | S24 | 39.95±37.36 |
| H2JA | D120 | 0.12±0.20 | S120 | 0.22±0.19 |
| H2JA | D96 | 0.00±0.00 | S96 | 0.07±0.13 |
| H2JA | D72 | 0.27±0.46 | S72 | 0.00±0.00 |
| H2JA | D48 | 0.00±0.00 | S48 | 0.00±0.00 |
| H2JA | D24 | 0.00±0.00 | S24 | 0.10±0.17 |
| IAA | D120 | 25.12±4.87 | S120 | 63.29±18.08 |
| IAA | D96 | 33.82±78.04 | S96 | 46.02±4.03 |
| IAA | D72 | 60.88±13.97 | S72 | 76.56±8.24 |
| IAA | D48 | 53.42±16.55 | S48 | 95.22±39.60 |
| IAA | D24 | 414.56±61.89 | S24 | 424.78±36.06 |
| IAA-Asp | D120 | 190.79±102.66 | S120 | 733.53±550.85 |
| IAA-Asp | D96 | 830.22±123.63 | S96 | 1469.00±329.22 |
| IAA-Asp | D72 | 607.63±176.09 | S72 | 552.92±94.27 |
| IAA-Asp | D48 | 1034.30±140.46 | S48 | 1030.90±89.28 |
| IAA-Asp | D24 | 1308.40±252.00 | S24 | 1324.30±463.85 |
| IAA-Glc | D120 | 27.40±47.46 | S120 | 655.93±578.66 |
| IAA-Glc | D96 | 204.14±353.58 | S96 | 710.78±115.24 |
| IAA-Glc | D72 | 509.87±809.43 | S72 | 77.49±20.64 |
| IAA-Glc | D48 | 248.16±429.82 | S48 | 70.12±61.27 |
| IAA-Glc | D24 | 88.20±78.13 | S24 | 432.28±527.35 |
| IAA-Glu | D120 | 73.77±14.91 | S120 | 250.29±213.44 |
| IAA-Glu | D96 | 212.63±134.31 | S96 | 167.37±32.45 |
| IAA-Glu | D72 | 127.52±52.56 | S72 | 153.54±27.86 |
| IAA-Glu | D48 | 176.59±46.56 | S48 | 294.17±98.07 |
| IAA-Glu | D24 | 187.96±76.42 | S24 | 341.85±262.03 |
| IAA-Leu | D120 | 0.25±0.44 | S120 | 0.98±1.70 |
| IAA-Leu | D96 | 1.27±0.44 | S96 | 0.94±0.81 |
| IAA-Leu | D72 | 0.23±0.40 | S72 | 0.93±0.17 |
| IAA-Leu | D48 | 1.69±0.59 | S48 | 1.67±0.60 |
| IAA-Leu | D24 | 5.15±7.81 | S24 | 5.78±3.66 |
| IAA-Phe | D120 | 0.45±0.30 | S120 | 3.26±3.49 |
| IAA-Phe | D96 | 2.26±2.30 | S96 | 2.13±0.88 |
| IAA-Phe | D72 | 1.90±2.27 | S72 | 1.42±0.35 |
| IAA-Phe | D48 | 4.42±0.90 | S48 | 2.16±0.93 |
| IAA-Phe | D24 | 6.27±7.97 | S24 | 7.93±2.67 |
| IAA-Trp | D120 | 0.00±0.00 | S120 | 0.38±0.43 |
| IAA-Trp | D96 | 0.86±0.14 | S96 | 0.84±0.11 |
| IAA-Trp | D72 | 0.40±0.35 | S72 | 0.73±0.21 |
| IAA-Trp | D48 | 1.23±0.36 | S48 | 0.88±0.78 |
| IAA-Trp | D24 | 2.36±1.98 | S24 | 1.44±0.16 |
| IAA-Val | D120 | 0.00±0.00 | S120 | 0.30±0.52 |
| IAA-Val | D96 | 0.39±0.68 | S96 | 0.00±0.00 |
| IAA-Val | D72 | 0.00±0.00 | S72 | 0.00±0.00 |
| IAA-Val | D48 | 0.00±0.00 | S48 | 0.33±0.57 |
| IAA-Val | D24 | 2.17±3.77 | S24 | 3.47±3.03 |
| IAA-Val-Me | D120 | 0.31±0.28 | S120 | 0.03±0.05 |
| IAA-Val-Me | D96 | 0.37±0.33 | S96 | 0.00±0.00 |
| IAA-Val-Me | D72 | 0.74±0.62 | S72 | 0.07±0.12 |
| IAA-Val-Me | D48 | 0.12±0.21 | S48 | 0.36±0.32 |
| IAA-Val-Me | D24 | 0.00±0.00 | S24 | 0.00±0.00 |
| IAM | D120 | 0.00±0.00 | S120 | 1.28±1.17 |
| IAM | D96 | 0.70±1.22 | S96 | 0.56±0.97 |
| IAM | D72 | 2.64±3.56 | S72 | 0.81±0.90 |
| IAM | D48 | 2.37±0.51 | S48 | 2.22±1.94 |
| IAM | D24 | 6.07±10.52 | S24 | 14.38±12.59 |
| IAN | D120 | 0.00±0.00 | S120 | 0.10±0.17 |
| IAN | D96 | 0.19±0.16 | S96 | 0.21±0.06 |
| IAN | D72 | 0.00±0.00 | S72 | 0.17±0.20 |
| IAN | D48 | 0.29±0.25 | S48 | 0.25±0.22 |
| IAN | D24 | 0.00±0.00 | S24 | 0.00±0.00 |
| ICA | D120 | 75.97±3.09 | S120 | 101.04±68.13 |
| ICA | D96 | 184.50±141.29 | S96 | 127.41±13.34 |
| ICA | D72 | 131.11±105.67 | S72 | 29.05±5.81 |
| ICA | D48 | 177.88±64.40 | S48 | 51.75±7.59 |
| ICA | D24 | 157.05±141.72 | S24 | 269.18±66.21 |
| ICAld | D120 | 27.05±3.05 | S120 | 86.67±39.89 |
| ICAld | D96 | 164.05±134.79 | S96 | 114.74±18.03 |
| ICAld | D72 | 84.19±68.77 | S72 | 59.27±6.57 |
| ICAld | D48 | 160.85±29.28 | S48 | 101.41±29.33 |
| ICAld | D24 | 320.53±399.56 | S24 | 534.87±294.50 |
| ILA | D120 | 95.44±34.28 | S120 | 119.79±78.38 |
| ILA | D96 | 241.09±128.47 | S96 | 266.56±108.76 |
| ILA | D72 | 86.82±61.35 | S72 | 185.77±22.59 |
| ILA | D48 | 158.54±78.67 | S48 | 113.16±32.88 |
| ILA | D24 | 162.02±280.63 | S24 | 478.02±137.86 |
| IP | D120 | 0.32±0.05 | S120 | 2.05±2.23 |
| IP | D96 | 0.67±0.39 | S96 | 0.50±0.20 |
| IP | D72 | 0.73±0.37 | S72 | 1.01±0.40 |
| IP | D48 | 0.71±0.14 | S48 | 0.89±0.27 |
| IP | D24 | 0.64±0.31 | S24 | 0.71±0.16 |
| iP9G | D120 | 0.30±0.52 | S120 | 0.00±0.00 |
| iP9G | D96 | 0.00±0.00 | S96 | 0.00±0.00 |
| iP9G | D72 | 0.33±0.58 | S72 | 0.16±0.28 |
| iP9G | D48 | 0.00±0.00 | S48 | 0.00±0.00 |
| iP9G | D24 | 1.72±2.99 | S24 | 2.25±2.00 |
| IPA | D120 | 7.94±2.69 | S120 | 7.24±2.77 |
| IPA | D96 | 1.53±2.66 | S96 | 4.89±4.24 |
| IPA | D72 | 5.50±4.87 | S72 | 5.72±0.20 |
| IPA | D48 | 7.46±6.47 | S48 | 4.76±4.12 |
| IPA | D24 | 3.33±5.77 | S24 | 27.66±24.72 |
| IPR | D120 | 1.46±0.59 | S120 | 1.97±0.51 |
| IPR | D96 | 2.07±0.45 | S96 | 2.26±0.21 |
| IPR | D72 | 2.49±1.10 | S72 | 3.33±0.40 |
| IPR | D48 | 1.70±0.91 | S48 | 1.63±0.25 |
| IPR | D24 | 19.21±3.87 | S24 | 27.96±1.79 |
| iPRMP | D120 | 7.21±1.57 | S120 | 5.25±2.13 |
| iPRMP | D96 | 7.42±1.67 | S96 | 7.75±1.12 |
| iPRMP | D72 | 9.57±3.38 | S72 | 13.39±1.59 |
| iPRMP | D48 | 11.15±4.70 | S48 | 13.19±3.85 |
| iPRMP | D24 | 15.82±11.13 | S24 | 21.16±9.23 |
| JA | D120 | 4.31±1.14 | S120 | 9.40±3.59 |
| JA | D96 | 8.31±5.86 | S96 | 11.66±3.53 |
| JA | D72 | 10.47±1.21 | S72 | 2.09±0.45 |
| JA | D48 | 6.44±1.54 | S48 | 2.32±0.94 |
| JA | D24 | 7.01±3.08 | S24 | 11.93±1.52 |
| JA-ILE | D120 | 6.96±1.72 | S120 | 17.56±14.47 |
| JA-ILE | D96 | 17.04±11.35 | S96 | 19.00±4.29 |
| JA-ILE | D72 | 16.98±15.34 | S72 | 7.24±0.64 |
| JA-ILE | D48 | 14.06±4.16 | S48 | 9.35±1.41 |
| JA-ILE | D24 | 20.27±6.18 | S24 | 25.72±4.31 |
| JA-Phe | D120 | 0.10±0.17 | S120 | 0.41±0.41 |
| JA-Phe | D96 | 0.39±0.18 | S96 | 0.35±0.13 |
| JA-Phe | D72 | 0.34±0.36 | S72 | 0.20±0.03 |
| JA-Phe | D48 | 0.30±0.32 | S48 | 0.42±0.13 |
| JA-Phe | D24 | 0.37±0.32 | S24 | 0.27±0.47 |
| JA-Val | D120 | 0.20±0.03 | S120 | 0.63±0.55 |
| JA-Val | D96 | 0.54±0.40 | S96 | 0.63±0.18 |
| JA-Val | D72 | 0.57±0.63 | S72 | 0.16±0.00 |
| JA-Val | D48 | 0.54±0.16 | S48 | 0.23±0.04 |
| JA-Val | D24 | 0.79±0.39 | S24 | 1.08±0.11 |
| MEIAA | D120 | 2.99±0.14 | S120 | 7.31±1.35 |
| MEIAA | D96 | 7.38±4.20 | S96 | 8.02±0.96 |
| MEIAA | D72 | 6.59±2.54 | S72 | 6.59±1.33 |
| MEIAA | D48 | 7.92±0.87 | S48 | 7.82±1.79 |
| MEIAA | D24 | 9.98±4.93 | S24 | 9.15±1.03 |
| MEJA | D120 | 15.62±16.16 | S120 | 9.20±10.35 |
| MEJA | D96 | 6.88±2.96 | S96 | 3.64±3.34 |
| MEJA | D72 | 6.98±2.69 | S72 | 3.91±1.17 |
| MEJA | D48 | 14.05±9.69 | S48 | 4.54±0.93 |
| MEJA | D24 | 22.27±17.67 | S24 | 21.30±9.58 |
| MeSAG | D120 | 1059.80±1835.70 | S120 | 1097.40±973.46 |
| MeSAG | D96 | 1995.50±444.22 | S96 | 1637.10±338.69 |
| MeSAG | D72 | 1025.60±917.57 | S72 | 289.05±500.65 |
| MeSAG | D48 | 1215.20±1183.30 | S48 | 537.68±469.94 |
| MeSAG | D24 | 0.00±0.00 | S24 | 823.03±1425.50 |
| mT | D120 | 0.14±0.24 | S120 | 0.25±0.43 |
| mT | D96 | 0.00±0.00 | S96 | 0.14±0.24 |
| mT | D72 | 0.17±0.29 | S72 | 0.15±0.26 |
| mT | D48 | 0.59±0.51 | S48 | 0.00±0.00 |
| mT | D24 | 0.12±0.20 | S24 | 0.00±0.00 |
| OPC-4 | D120 | 29.65±26.22 | S120 | 51.59±2.86 |
| OPC-4 | D96 | 48.50±30.42 | S96 | 45.66±21.57 |
| OPC-4 | D72 | 43.30±75.00 | S72 | 0.00±0.00 |
| OPC-4 | D48 | 35.96±34.88 | S48 | 0.00±0.00 |
| OPC-4 | D24 | 23.98±41.53 | S24 | 15.78±27.33 |
| oT | D120 | 0.53±0.51 | S120 | 2.15±2.76 |
| oT | D96 | 2.70±4.16 | S96 | 0.04±0.07 |
| oT | D72 | 1.22±2.11 | S72 | 0.62±0.76 |
| oT | D48 | 1.09±1.89 | S48 | 0.10±0.18 |
| oT | D24 | 14.41±24.95 | S24 | 8.63±7.18 |
| oT9G | D120 | 2.87±2.46 | S120 | 1.12±1.24 |
| oT9G | D96 | 1.51±1.45 | S96 | 0.00±0.00 |
| oT9G | D72 | 1.11±1.92 | S72 | 0.84±1.45 |
| oT9G | D48 | 0.81±1.40 | S48 | 0.00±0.00 |
| oT9G | D24 | 2.53±4.38 | S24 | 1.37±1.23 |
| oTR | D120 | 7.33±7.04 | S120 | 0.24±0.41 |
| oTR | D96 | 4.50±4.12 | S96 | 0.00±0.00 |
| oTR | D72 | 2.79±4.84 | S72 | 0.27±0.46 |
| oTR | D48 | 1.54±2.67 | S48 | 0.00±0.00 |
| oTR | D24 | 20.00±34.64 | S24 | 15.72±22.14 |
| OxIAA | D120 | 54.84±6.58 | S120 | 97.72±26.81 |
| OxIAA | D96 | 110.86±57.93 | S96 | 99.78±12.50 |
| OxIAA | D72 | 90.29±36.28 | S72 | 117.39±18.00 |
| OxIAA | D48 | 128.74±31.82 | S48 | 141.98±26.18 |
| OxIAA | D24 | 268.34±258.31 | S24 | 385.51±184.61 |
| Phe | D120 | 13589.00±2576.50 | S120 | 8601.80±2552.10 |
| Phe | D96 | 11859.00±1591.80 | S96 | 10996.00±640.17 |
| Phe | D72 | 11884.00±2858.90 | S72 | 12133.00±1266.00 |
| Phe | D48 | 8995.10±1066.60 | S48 | 12649.00±1323.90 |
| Phe | D24 | 15735.00±7467.40 | S24 | 19765.00±8283.80 |
| SA | D120 | 1629.50±81.75 | S120 | 517.66±163.91 |
| SA | D96 | 1041.60±62.08 | S96 | 443.29±67.67 |
| SA | D72 | 1215.80±438.10 | S72 | 309.49±43.22 |
| SA | D48 | 447.29±176.03 | S48 | 292.47±35.18 |
| SA | D24 | 700.10±752.10 | S24 | 1286.70±149.41 |
| SAG | D120 | 228.96±68.39 | S120 | 88.79±40.51 |
| SAG | D96 | 220.56±120.38 | S96 | 124.00±22.94 |
| SAG | D72 | 344.88±404.68 | S72 | 98.75±36.80 |
| SAG | D48 | 54.47±94.34 | S48 | 91.19±15.07 |
| SAG | D24 | 96.69±93.11 | S24 | 167.86±33.19 |
| t-CA | D120 | 113.58±37.85 | S120 | 148.48±88.99 |
| t-CA | D96 | 170.98±70.75 | S96 | 211.49±21.45 |
| t-CA | D72 | 161.40±137.32 | S72 | 233.17±83.00 |
| t-CA | D48 | 133.47±13.58 | S48 | 276.44±67.45 |
| t-CA | D24 | 979.09±424.40 | S24 | 1484.40±185.30 |
| TRA | D120 | 149.63±100.00 | S120 | 24.47±42.38 |
| TRA | D96 | 76.33±132.21 | S96 | 0.00±0.00 |
| TRA | D72 | 207.43±317.74 | S72 | 52.78±5.17 |
| TRA | D48 | 53.25±48.75 | S48 | 104.61±5.31 |
| TRA | D24 | 99.90±17.67 | S24 | 84.02±72.91 |
| TRP | D120 | 4361.80±891.87 | S120 | 8502.60±3212.90 |
| TRP | D96 | 6629.00±1830.10 | S96 | 6457.20±574.40 |
| TRP | D72 | 8069.40±2781.90 | S72 | 10572.00±1714.10 |
| TRP | D48 | 6803.90±762.93 | S48 | 10859.00±1256.20 |
| TRP | D24 | 11661.00±5356.30 | S24 | 14198.00±8009.00 |
| tZ | D120 | 0.37±0.06 | S120 | 0.03±0.02 |
| tZ | D96 | 0.07±0.03 | S96 | 0.03±0.00 |
| tZ | D72 | 0.19±0.09 | S72 | 0.17±0.07 |
| tZ | D48 | 0.00±0.00 | S48 | 0.06±0.01 |
| tZ | D24 | 0.20±0.08 | S24 | 0.00±0.00 |
| tZOG | D120 | 574.14±50.38 | S120 | 417.06±223.23 |
| tZOG | D96 | 484.31±66.68 | S96 | 487.27±40.25 |
| tZOG | D72 | 576.45±160.10 | S72 | 389.13±82.07 |
| tZOG | D48 | 611.46±51.56 | S48 | 534.24±46.12 |
| tZOG | D24 | 418.93±228.01 | S24 | 353.81±258.63 |
| tZR | D120 | 0.31±0.10 | S120 | 0.08±0.07 |
| tZR | D96 | 0.11±0.09 | S96 | 0.05±0.04 |
| tZR | D72 | 0.22±0.12 | S72 | 0.10±0.09 |
| tZR | D48 | 0.11±0.19 | S48 | 0.04±0.06 |
| tZR | D24 | 0.86±1.12 | S24 | 1.20±1.14 |
| tZRMP | D120 | 16.09±2.79 | S120 | 3.60±6.23 |
| tZRMP | D96 | 4.57±7.91 | S96 | 0.00±0.00 |
| tZRMP | D72 | 8.89±9.14 | S72 | 15.62±1.88 |
| tZRMP | D48 | 14.55±12.68 | S48 | 18.54±8.92 |
| tZRMP | D24 | 20.49±17.88 | S24 | 0.00±0.00 |
