# Supplementary material for: Multi-omics profiling reveals single-seed mutants of Ephedra saxatilis as dominant variants in high-altitude Xizang
Source: BMC Plant Biol. 2025 Aug 22;25:1118. doi: 10.1186/s12870-025-07153-x (PMC12372293; doi:10.1186/s12870-025-07153-x)
Supplement: Supplementary file 4 — Supplementary Material 4. [file 12870_2025_7153_MOESM4_ESM.docx]

23 Transcriptome gene ID and gene sequence in fig.8

| GeneID | Sequence |
| --- | --- |
| Cluster-70657.0 | TTGAATTAAATTATAATCGTTAGTGTCTTAGTATAATGTGTGTGACCACCACAAAAAATAGATACAAAAGTCAGAGACTAATATCAAGTTGAAACAAACACTTTCAACATTTAAATTATTCTAAGCTGTCTTAGAGCCTTAGAGGCAAAACACCAGAGTCACAAAATGAGCGAACATGATGGCACTAGACATCAACCCAACCTAAAGTAACTACGCAAGATAACTTACTATAGCCCAAAAACATGAACAGTTATTCAAACATTTATCCAGCAAGCATTTTATGAACATGTCTAAACCATCAAGGAGTTGGCAACGTTCCGGCCTTGTTGTTGCGCACTGCTGTGTTCGTCTTGTTGCGTTCAGATAACCTTGCTTTCACTTTTACATTGCAATTTCGTTTTGCCTTGGAAAGCATTTTCCCATTTCCCATCACCTTGCCATGCCTTACTTTGCCAACTACTACCACTTTCTCGTCTCCACTACCTCCACCGCCACCGCCTCCTCCGCCGCCTCCGCCGCCGCCTCCGCCTCCTTCATCGTCGTCATCATC |
| Cluster-101942.3 | CTACAACACTTTTTGGCTATGCTGAAATTCCCTTTCCTTTCATTTGAGAACTACGAGAATTGATTTCACAGACAACTAAACAAGCAGAGTTTAACATAAAACTATACCATCAAAACACGACTTAAGGGTACTTGAAGTACACTCTTTTCTGCCCCTCCTCCTCCTGCGTATAGAGCTTCTAAGAAAAATGGATGAAGCTAACGATATGAGTCAATTTTGGGGAAGATTGTTGAGAGAAGATGACACAAACAATAGCAGTCTTGTCGATCAATTGCTACAACAATATCAGGAAGAACAAACTCTCCAACTCAATCTCAATTCACATACTCTCTCCAAACCCTTGAATTCCATTGACAGTTCTCTACTCTCTCCGGCCTCCGTAGACATTGCCGGCCTCGATGAGGACCTTGCTCGCCGCTATGCAAACCTCAAAAAAAATGAGGGCATCCATAAAAAATCTGAGGGCATCCACAAGGGTTCCCATGAAAATTCAGGCAAAGTCCAGAAGAACCATGAAAATCCATTGAATAACAACAAAAATTCAGGCAGTTTTGAGGATACCAACAAAAAACCAGGGAATTTCAATGACGCCCTCAAAACTTCCGAGGGCACTCACGAAAATCTAGGCAAATTTGAGGATACCCTTAACAAATTCCAGGATACCCTCAAAAAAACTGAGGGCACTCCTGAAAATCCAGGCAAAATCCAGAACCCCCATGAAAATTCATTGAATTTTAAGGATAACCATGAAAATGCCTTGAATTTTAATGTTACCAATGAAAGTTTTAAGGACACCCTTGGTGAAAACAAGGGTGAGAAAATTAGGGATGACCATGGAAAATTTGTGCCTGTTGATGAAGAGGAGTTGCAGATTTTGGGATCAGAATTGGCATCCCGCTTCGCTGCCTTGAAGGCCAATTCTTTTAAAGGTTTGGAGAATCCTACAATTACCCTGGAATCCAAAGCAGGGAATTCTACTTCAGAAGTGGAGACTAGTTTGTTTGAGAGGGGTCCCCAGAATCCTAATCTGGGGAATGCAAATGAAGATGAGGTAAAGAGATTATTGGCTTCTGTTCAGGATGCCATGAGGCTTGTACAAGATAAAGTTTCGGGGAGTTCGCCAAGGGTGGCTTTTGATGATGAGACTGAGAGGGGTGGTGATTCAGAAGTGGAAAGGATTGTAGAATGGGCAAAGGATGCTGCTAGATTGGATCCCAGTTCGGAGGAAGTTGAGTCTGATGGCGGATTTGAAGATGATGATGATGATACTTCAGATGAGGACGAATCGAGTGCGAATCATAAGAAGAAGAAGAAGTTAAGCTCTAGATCAAGAAAAGAAGATAAGTAGGTTGGTATATGTGTACATGTTAGAACCTTCTTTACATGGTGATGTTTTTGTTACTTTTGATGGTTGTTTGCTATATCCAATTTAGTCTAAGATTGGATTATAGTTTATGTTCACAGTTTTTAAAAGCAGACATCTTTTGTTTCCTCACATTGTGATCTTTCCTATCCCCCGCTTTTATGACAAGAAGTCATTTTTAAACATTGTGAAATGTATTGATGAAAATTACTACTTCATGTTCTACAATTCTAAACCTTTATGTTTTACAATTACCCTTTGGAAACAAAATGCAAATA |
| Cluster-61487.7 | CAATAAAAATAAGGCCAATTGCATGCAATCAGCTGCAATCAAACAGACCCTTAGGGTTTTGCAAAAGATAATTCAATTGGGAAAATGAGTTGTATACAAGATAGTTCAATTGGTAAAATGAAACCTAATTCTCTTTGAAAGGTTACACTTTTGGCATAATCATTTTGTAGAGGGGTGACAAATCCAAATCAAATGTGCCCAGAGTAACAACCCTTCTTTGTTACAGACATCTAATTCGTTTTAAAAGTGTCAGGAACATAATTGGAGTATTTACTAGTTAACACATTCTGACATTCTTTTGCCCCGAGGAGTCGAGTAGTCTGCATCATGTGCCAATCTTCAGAAAGGGTAAAATACTAATCAAAGGTTCCATAATAAGCATCGTGCCATTGATATCCATTGCTGGCATTGGGGTTACAGACTGAAGTGCCCCAGCCCAGGTTGTGAATAAAACTGTAAACAAAACTTTAGATATGCTTGATCACGTACTTCAAGAAAGTAATGCTTAATCATCTACTTGAAGAACGCTATTCCATGCCCTGAGATAAAACTGTAAGCAATATTTTGGATATGATTAATCATGTAATAGGCATACTATTGATATGCTCCCCTAGAAAATAGTCAATTTGCTCAAATGTGCACAAAGCAGAGTTCTTTAGTTTAGATTGTAAAACCAGGAGTAAAGGTATCCATATGCTTAATCTTTCTATTTGGAGGACGATTGTTAATTTTCTTGGTTAAAGCAACAATCAACATCAAGCTGCTAAAACTGATGCACACACATTTTGCTTCATCTGAATGTTAATGGCATTTGTACCCTTATAAAGAAACTAAAAACAAGATGTCAAGTAAAGCCTCGTGATACACAGAACCCCGAGGCTTCCTTTAAGCAGATATTTATCGAGTTCATACAATAAAAACATAGCCTCGGTGTGCAGAAAAGAAACCTATGTAAAATCAGCCAATCGGATAGGTTTTTATCTGCACACTGAGAAAAGATGAGATGCCTTTTGATAGATTTAAACAAAATAGAGGTACCAATCTTCAGACATTACTGTAATTTAACATATTTGGAGATTATATTGGAAAACTTGAAAAAACTGTTCTTCAGATAACATTTTTGTATATATGGATGAAGAGGATTCATCATAAACAAATGTGCAAGATAGAAGGCATAGTTTTTGACCTCATAAACCCTTTAAATCCATGGTAATCCATTGAATTAAAGTTTCCATGTTCCATGTCATGCTTATCAGCCACCTCCAATGTTCCTCCGTGAATTTGATGGCTATTGCTTTGACATACGACAAAAGCATTGAAGAAAACGTACCACCATTACGCTTCATTTTGCCACTGATCACTCATTGTTCTCTCCAAATGCGTCCGGTTACTCTCAATTTTAACTCTTTCTTGTCTCCACCGGAGAAAAATAAAGCCATGTTTCTGTGTATAATTAGACCATCATGGCTATCACGGACTTTCCTGTGGCTGTCTCTGATGCTCCTAGGAACACCTTGCCAGAGCAACTTCCGACCATTTGCTCCAACCTCAAGACTATAGCTAAAGTTTTTTGCTTCATTGTCATCTCCCATAAATCTAAGAAAGGCGATATACACAGGTTCGCCCAGTTGAAAAGCTTCAAAATGTAGACAAAAGTACTGACCAAAACAATTGAATACTGTTAACATCCAGGTCGCATTCTCAACCTCATGCGGATTTGACTTCACATATCTGTGGTTGAATGTACTGCCAGTGTGCATATCCACTTTATGGTCATCTCTTAAATGAGCAACCAAATATTGAATATCGCCAGTGACAGTACATTCTGAACCTGCATAAGGACAAATGTATGGCCTGAATGAGCATTGAGCTTCATGCTTAAGTTTGCTGTAGTACGGAAATATGTCCTGGCAACCATGAACTTGGTATTTGCAAGGAAGTTCTAGGGATGCTGCCACTTTTTCCAAAGCCAAGCATCTTATATTCCCAAGCTCAAATCTGCAAGTAGGGCAACGGTTCTGAACTCTCACTTTGCAGCTTGAGCAAAGTGTATGGCCATTAGGACACTGATGAATAGGAGGATACATGGAATTTGTACACACTGGACATTCCAGCAATTCATGAACACTGCTTGTAGTTGGGACATTGGATTTTACAGTATGCCGACTTTTTGCTTTGACCTCACCCACTTCCACTTCCTGTGCAGGCTCAAGGGCCGCAGCTAAATCTTGAATGTCAAATGTACGAGTGTTGCAGGAAACAGAGATATTTCTTTCAGCCATGATTCTTTATATTAATTACTTCAATGCCAAACACTACTCAACTTGATATTACCACAATGATTTCTCCATGTCTGTGATTGATATCATAGTAGTGATGATGATGAACATATTTTTTT |
| Cluster-80148.0 | CAAGACTATTCTTTATCATCAAAAGTAAATATCCATACTTGTAATAGAAGTATAAACTTTTATTTAATCATAGAAGGAAGATAACTTCCGTTGCTAGAAATTAGAAAATACCTCAAATACAAATCAATTTGAGATGTCACCCAAACCTCCCATAGACAAACCGAACAAAAACACACACTACCTAAAACAAAGGGGCACGCACTCAATCCACAATGCTTTATTTGCAACAAAGCCACCATAGGTGCATGTAAAAAAACTATCAAGGTGGGACCGTAGATTTGGTTCCATCCATAACGTCGTTCATGGCCAAACTCTTCTTGAGCCCTTGATTTGCCTTTCCGTTCATTTCCGCAATGCCTTTATTCTTCCTAGGTTTTGCCTTCTTAACCATTTTTTCGTCTCCACCACCGCCACCGCCGCCACCACCTCCGCCTCCTCCGCCACCGCCACCACGTTCGTCGCCTCCATCATGACCTCCATGACCATTTCCATGTCCGTTACCATGTCCTTGTCCCTTACCGTCATCACCGTGATCTCCGTCGTCGCCGCCATCTTCACTGTCCCCATCGTCATAGTCATCGCCGCCACCGCTCTCTACAGCTCCGTTACCGTCACCGTTTCCTCCTATTTCACCGATGCCTCCATCATCATCATCATCGTCGCTACTCTCCTTCACCGTGTTGTTCTTTCCAGCATTTCCCGTCACTATTTCATCTTTGTAGTTGGAAACCTTCCTAGCATGGGCAGTTGCTGCCACAGAAACCAAGAACAAGAAGGTGAAGACAGCCAATACCACAAACTTTTTCTTCATCTTTTTGGTTGGTATTGTTTCATGCTTGGAAGAGATTATTTATAGTAGGTTTCACAA |
| Cluster-17514.6 | CTTTTAAGACTAAAATAAACTATTAAAAATTGTCATTGAAGATTATACATATATATTTGCAAACTGTGAGTTAATAATGAAAAATACTTCTACTTTCTTTGCTTTTTAATTATAAATACATTTTTTGGGCAGAAAAGTATATTGATGCAACCTGTGCTACTTTTAAATTCTTACACTGATAGATCCAATTCCAGCATGAGTTTTTAACTTTCTCTAGTGTGAAAACCATTAGCTTGTCGTATTCAGTAAGATGGAAACAACCAATGTTTCAACACACAGTTGTATCTCCATTCACATAGTGAAATATCCATTGAAACCTTTATTCTACAAGAATGGAAAAGATGAATAGATTAACGCTCCAGCGTATAGCTAGTATTGGAGCTCACAGTTGACCATGAGAGTTGCTAATCCCTAGGCTTTTGAATTGAATATGGATGTTGAGAAGAGGTACCAGGTCTCTTATAAGGATATTTTTTCAAAATCAATAACATCACCAACTACTCTGGATGACTATTTTCCCATTTGTGACCTTGAAGTTGTTAAACAAACACCAATTCTGACTATGCCCACGAAGAAAACTAAACAAAATCAGTCTGCATTCTTTTGCTCCCTATCCTTTAGCTTTGTCTTTTTTTTAGGCTTGAGACTTTGTTTCTTATCTGTACCAACAGGAATTTCATCTTCCAAGGACTCTAAATCATCTGAAGTTGTAGTATGTCTTTTAGATGATTTTTTCCTTTCATCTCTGTCATGTTTTTTCAATTTCTTTTTAGATGATTTCTTTTCCTTGTTAGCAGACTTCTGGTTTTCATCTCTGAAATCTGTCTCATCCAATTCAGTCTCTTTGTGTTCATCTATTAAATCTGTATCATTCAATTCAGTCTCCTTGTTTTCAAGAATCTTGTCAATAGCACCTGCGATAGTTGATGCCGGTGTTAAATTGGGATCAAGAATAACACTCACAAGCTGTTGTTTTAAGAAAACTGATGCAAGGATTTGCCGTTTTTGTGTATCCCAAATACGTAAATAGCGATCCAGTCCACATGAAGCAATTAATGTACGTTCAGGATGCTTAGATATTGAGCGAATGCTGCCAGAACATTTTCCTTTGAAAGCACCCAATAGCTTTCCTGTTCTCATATCAAAATATGAAAGATCACCAGTTCCATTCCCCACATAAACAGTATGACCATCTGAATCTTCCTTGATTACCTTTATTGGAGTGTCACCAAAATCAAAAGACAGAATAGGTCTTCTTTGCTAGTTTGCCTCCCTCACTGTTTTCCATGCTGGATTTGAATAGGTATTTCAGTTGTTTGTTTTTA |
| Cluster-21748.0 | TTTGCTCTGAAGGAGAATTTCAGATCTGCGAAAGTCATTGTTTCTCTGCCTTGCAGCTTACATAGACACGTCTCATATTCCAGACGGGCAATGGAGGCACAACAGAGCGCAATAGAGACAGACACAGACGGGAGCTCCCTAATCCCTGGCTTACCTACCGAGATTGCCCTAGAATGCCTGGTGAGGGTTCCCATGACACACAAGATTTTGCAGAAAATGAGACGTGCGTCACCTGCCTGGAGGAGAGACCTGTCCAACTTCGACACATCTGTAGTTCTGAAGGAGAGACGCAAAAGAAACCTGCAAGAGCCGAGTCTCTTCCTGATTCCGTGCATCGACAGACACTCTTTACAGCCACTAAGATCTGATAGTATGACGTGCACCTCATATAAGTCATCTATTCTTGTCTTGGGAGGATTTAGAGATAGAGAGGACGAGTTCAATCGGGTGGTTCCGGATTTCTGGATGTTTGATGCTTTGTCGTTTGCCATTACAACAAAACCTTTCATGATTCCGCATCGTCAAGTTATGGTGTCTCCCATTCTCCGTGACCATCTCTTTGCCGTAGGGGGAATTGTACAATTTGACTTTGGTTGTAATGAACCCGATATATGTAATGAAGTCAGTACGTATACATACACCTCATATATAGTTCACACTAAAAAGTTTTGTCCGTTGAGTATCGTATAAATATTTTTTTGGCTTATTAATTGGCCATTCGTACCCCACTAATTAGACCAGTTGTTGTAGATACTGTGGAGAGATTTGGTTTAGCAAGTCCAGATCCAGCGTGGTCTGATTCACCTGATCTGCCTGTTAAGACGGAACCAGAGAGAAGCTCGGTGTACGCGGAAGCAGAAGCGGATTCAGATGAGATGCTTGTTTTACCGGGTGAGAGTGATGAATTGGTGTTATCCTATAACTCGCAAGGAGCTGATGCAGGTACATGGACACGGATTCTCCGCCCGGAACTGAAGAAACACATGTCGAGCAGATGGATGTACCATAGTGGCGAACTCCGTCTCCGTAAAGAAATGGTGGAAGGTGCCGGTGCTTTCAACCAGCATTTTCCTTCTGGTTATTTTCAAGTCCATCAAGACTTCGCGAGGGCGCTGGTGCAGCCAAGTGGCAGTGGTTACGAGCATCCCCTGTTTTCAGACTCATTCAAGAATAACAGAGACCTGCAGCTATGTGATCATGATGCCCATACAGAGACCGCCTCTATCAGATCTGTTGGAAATATACCTAGGAATGCAGTGTACCATCATCCAGATTTCATAGCCGATTTTCTGCAGTCTACCATGGTATGCACTGTTGCTGGCACACCTGCCATGAGAGAGAAATTTCTTTTTCCAACTGATGATAGCCTTGACTGTCTTTTTGCAGTCCTCTTCTTTTAATGTTTTTCTTCCCTTGATATCTAGATGGTGCAGTGAGTGCTGCTTGTTCGATGATGCAAGATGCTTGCTCTTTTATCTTTCCTGTCCGCTGATTTTTTTTTTATAAGCCATTTTACCCCGTATATTACATTGCTAAGATTTGTTAGTGCTATGTGTGAAAAAAGCAGCAGCAAAGCATCAACACAGATTCTTTGGTGAAAGTCTTGCTTCTTATTTTGTTGAATGATGCAAATGTTGCAGATATTGTCCACGTATGCTTATATTTTCACTACTACAAAATCCTGATATAGAGACATTATATAGAGACAATTTAGTC |
| Cluster-87331.2 | TTTAGAAAATCAACATCCTATTCCTTTGAATAATATATGAAGAAAATTAAACAATTATAAAGGATATGGTGGTTCTTAAGCAAAATATCAGATAGTTTAAGCTATAAGCTAAACGCTAATACATAAGAAACTGATATCTCCAAATCCCTTTCTGGTATTACCACACTGTTTTGTGTTTTATACTATTTTTTGTTCCCTAGAAAGAAGAGACAATGTAATGATGATACAGTGTAGCAGCAAAAATAGAGATGTTATAACAGTTATTAGGGTAATACAAGAACCACTAAAGGGATCTTAGGATATTCATTCCAATGACATACCTAAAAAATTTGAAGCTTTCTTCATGCACTAAACTTATTGATTAGATGATCTCAACAAATCCAAATCAAATGGACATCATATGCTCAGTTTGGAGGCATGTTATTCTTTGATTTTGTTTCAAAGAATATTCAAGTAAATTTGTCATATGTCCATGTTTTAATAAAAGAGGCCAACTCAAGCATCATTGCTATGTGTAGTATTTATGTTATCAAGCAACCTTGCAACCTGTGCCATAGTCGGTCTTTCCATAGTACTTTCACTCAAGCATTTACAGGCAACATCAAGGGCATGCTTTATCTGTTCTAATTCGGGCGTATTTCCTATGTGTGGATCAATCACATCCTTCCAACATCCAACTTCCACACAGGCTTTTACCCATGAGACCATGTTTTTTCCATCTTTAGCGGGAGATTCCTCATTGGGCCTTTTTCCCGTTATCAGTTCAAGCAACACCACACCATAACTATAAACATCTCCTTTTTCAGTTGCTCTTCCTGTTTCGACATACTCTGGTGCTAAATAGCCAAAAGTTCCAGCTACATATGTAGACACATGTGATTGATCTGGACTCATTAAAGTTGCTAAACCAAAATCAGATATGCGAGCTTTCATTTCTTCATCCAACAATATATTACTGGGCTTTATATCTCGATGTATAATATGTGGAATACAATCATGATGAAGATATGCAATCCCTCGAGCAGCACCTAATGCAATTTTGTATCGTGTAGCCCAACTAAGAGGTTCAATATTTGAAGATTTATTATGAAGAAACCTATCCAGACTTCCATTCTGCATGAGGTCATATATCAAAAGATTCATACTGGAAGCTTTGTAGTAACCACGAAGGGTTAGAATGTTCCTGTGCTTTATATCACCCATTGCCTCGAGCTCTCTTTCAAAACTACGGTCCTTATCACTAGTATATCGCAATAGCTTCTTGACAGCGAATGCTGTTTTTTCATCATATACTAATTTGTATACTGTACCAAAACCACCAGTTCCAATAATGTCTTTACTAGTCAGTGTCCTTGTTTTCCTTAACAAAACTGCAGTTGTGATTGTACTTTTTTCTGGCCATCGAAA |
| Cluster-43243.33 | ATTGCTGGTCCACGTGCTCTAGAGCATATTGTAGATGTCATTTTATACATGGAGGCAGAAGCCTTTCTTTCACATTGGCTAATTAGACCTATCAAGCTTACAAGTAAGCTTGAGATTTAATTTTAATTTTGTTGTAAGGTGAGGTTTGAGTGATTATGATGTGCAGGGTGGTCCTGCTTACCTAAGAGTGGATGATGGTAATAAACATGGCTGGCCCTTTACCAATGGGACTCAGAGCAATAACCTGTATATATTTTGGAGTCTTTTTATTAGGCCACAAGGAAGGTATGGTAGAACTGTTGCATTCAGTTTTTTTTTTAAACAAGTTCCAAAAATAGATATATCTGTTGTGCTTTCCAAAAATTCTCATGGTTATTTAGAAACTATCAAGTTGATGCTTTCAAGTTTCTATTCATAGATATGAAAAAAGAGAGCTGGGGAAGCTTGTGAGCAGATACTCGGAGAAGGACCTTTAGGAAATTTTGGGAAATCGAAGTCAATCATCCACAACATCAAGTACCAAAGAAGAACAACCTGTAACAGATAAAGCCAATGCCATACGAGATGATAAGATAGAAACTGTTGATGATGAGTATTATGAAGAAAGTTGTTTTGGAGCTGCGGATATAGCACTGCTATCCAGTGCTCAGTCAGAAGATTGGTGGGGACATAAGCATGGTTTTGTTTCAGGTGGATTTCTTGGTGCTAAATCTAAGGTCAAGAAAATTCTATTGCCTCAAAACGAGAAGAGAAATGAATTAACTGATGGTGAAAAATCTACTGCTAGATTATCTACAAGTCGAGCTACTTTTTGTGAGAAGGATCAGGAGGACCTTTACAACCTCGTACAGGGTAAAGCCACAACAGGCAAACAAGGACTTGGGATAGGTGACAAGCCAAAGAAAATTGCAGGAACTCACTGGAAAGGACAGAAAACTGTATTTGATAATGACGACGATAAAGATAATAGCTCTGAAGTTGAACATAGTGAAGAGGATCTAAAACAAATTGAAGATTCCAAGAACAGAGGGGATGCAGAGGATATTTCAAATACTAAAAATGTGAAAAGGAAAAGAGTTACAGAAAAGACTATTGATATCGATATCCTTAGGCCTAATATGAAATGGAAAAAAATGTGCAAAATTATACTTCAGCAGGCTCCAGGACAATCAATGAAGATAAAGCATTTAAGAAAGCACATTGAGGCCCAATCTAGTACCATCTTCTCTGACTGTTCTTCGGGCCGTGATGCTACTCTATTACTGAAAAGAAAGCTTGAACGCAGCTCAAAATTCTTGGTTGAAGGCAAGGCAGTGTCACTTTGCAAGACTAGATGACGGGTGACTTGTTGACACCTCATTTTTCCAACTAGATGATGGGTGTCTCTTTAGAGTTTTTGTTGACGCCTCATTTTTTCAAGAGTGACTTTTTCTGTTGTACCCATTGAAGCCTCATTCTTTTAATATTATATTATTTTTCAGATCTAATGTAGTAACAATGACCAACTAAAAGCATACCACTATTTACAAGACACAATGTAGTGGTTATCAGTTTTGAGAGATTGTTGGTAATTAAGGATTTTTGTTGAAATTTTAAAGATACAGGATAAAAAAGCTCAAGAAATCGCATAAAATTGTGATTTTAATGAGTGTATATTGAACATTATTAATTGACATTATTATGCACTTTTCTATTGTATGCATTGAAACATTTTTCTTTCATGTTGTATTACTTTCA |
| Cluster-89147.3 | ATATGAAGAAGAAATTATTATTTGTTTAAATCTAAACTAAAATAAAATAAATTAAAAATGGAATTATTTTTAGTTTTATCCATTAAGATTTGTACAAATAAGACATAATTTTATTAGAAAAAATCCACCAACAAACGTTATGTTCCTCTGAATCACCCGTAAACATCGATGGTGAGTGTTGAGATAGCCTCGATTTCCTACCGATGGCAAGATGCACAGGTTGCAGTTATCAGCCACTAATGTAGCCACTATCATTGCTTCCAATGCAGCCTCCAAGACACAAAGACTGCCCACTGATTTGATCTCCGTTCCGAACCAACAAGTCAATCCGAGACTTGAAATGGCTCTTCATGATAATGTTAGGCCAAGTGTATTGACCTTTCTTGGGAAAAGAAGCCATGCCATAACAAGAAGCCTTTACGGCATCGATGATGTTGGTAAAAGAGTAGAACCGTCCCATGTCTCCCTTGTTCTCCCTGAACCATCATGGATATCTCTCTCCATGTACCTCTCAGACCACTCTAACTAAGCACCGTATTCAATAGGATGTTGTAACATGAACACATCCTTAAATGCATTTTTACTATGAACCCATGTCAAGAAACACCGTTAGTAAGAGAGGAGCATGAGCATGCCACCACCATGGTGTAGAAGTGAGATGTTGCACCAATGGAAAATGATCCAAAAGAGATTGGGAAGGTGGATATTCATGAGAAATTGCAGATCCAGACATGGGAAGGGAGAGACGCTCTTAGAAGGGTTCAAGATGTTGTTAGAGCGGCAAGCTCGATGACACAGACTGAGAGGACGGTGAAATGGGCAGAGAATTTAGGGTGATAGGATGTAGTAGCAGAAGCTCAGCAGTGAGGTGAGGGTTTGAGGAACTTAGAGCAGTGAAGGAAGGTAGGAGAGGATGATCTGGGAGCACCAACAATGGTAGCACCTGGATGGAGGGTGTAATGCACTTGACACGGCCTTGGTGGAGGTTTGGGAATGTCAAGAAGCTGACACAGGACATGGCAGATTGAGCATGAGAGGCTGAGGGGACGGAGATGAGGGTGTGAAGGGAGGTCTGCAAGAGGTTTGAGAAAAGTGGCAGAAAGTGGGGACGTAGACTTTGAAAAGAGAAAGTAGCCAAAGTTCCTAATTCAAACTTAAATCCTAATCCCAAAATCCCATTAGTCCATACGTTCCACACCTATGCCATCATTTCTAATATCACTTGCCACCATATGTCAATTAACTGCCACCCATCTTCTTCCAGCATGACATCTGG |
| Cluster-57528.0 | CTGTGTTATCTGTCAATTGCTTTGTCTATCAAAATAATTAAGAGTGTTGCATTTTCTAATTCTTTTATTTTTGCAACATTCATAGCATCTAATATGTTAGCCAAAACTTATGTAAGATGATACACAACCAGTAGTATTCATGAAATTTGGATAGAAAAAAAAGGAAGCCTATAGGGAAAGAGTCCACATATTTGGACATTTTGTCAAACATTTGAAGAAAATCCCCACCCAGTATTAATCCGGTGTTTTCTTCTACGGGTCCTAGCCATGGAATCAAACCTAGCTTGCACGCATTTGATGCGCCTTCGAATCAGCACCCTCTCGCTGATCACGGTGCACCGCATCACCGGCCAAACCCTGATTTAAAGTAGGCATTGTCTACCATTGCATCTTCTCTCCCTTCAATTATGATCATCCATGTACTGGATCCATCTCTCCTTGATTCGGAGACCATCCTAAACCATGTAAACTCCATTAGATTTTAGACCTCAGCCTCCAATGAACGGAAGCCTACCAACCATGGATCCGTTCATTGCTGGTCGCCTACCTTCACGCTGTGCTCTCCAGCCCACGGATTCACCAACTCCACCCTCTGCCTTGCTTCCCAGCCGACTTCGGCATCAGTCCTACCTTTCCCGAGCTGTAGCGACGGCGCCGCCTAGACTCTGCCATCTGAGCGATCTGTTCTCCCCGCCCTTGAGCAGCCGTCTGCTGATGCTCGCCGCGACCGTGTTCACCGATCCGTCGCTGCTCCATCTCCGAACCCGTTTCTCTCGTAGCTGCCTGTCTTAGGCTCCCTCTTGCAGCCAGCGAGCTCTTCTCCTTCCGTTCATATCTGCCCCTCGCTGCCCTGGTGTGCCTATTACCATCACTCGCCCAGTCATGTGCCTTTGAGGACTGCCCTCGCATACACCTGACCTTGGCTTATCCCGTGTACCCTCTGTGCTCCCAGTGCACAGCCACGGACGCCCTAGCCCTTCTGCCATTCGCTGTGAGCCACCGAGATCTCCATCTTAGCGCCACTCTGCTGTGCATGAGCAGGGAAGAAACTCCTACTTCTTGGGCCTTCCAGTCTGCCGCGCCCTAGTCCCGGATCATTATCTGCGCCGTCAAACATCTGGCCCTCCTTTGACTCCGCCATCTCTCTACCGCCATCGACTGAGCCTCGGTATTCCTTCTTCCCTAAGGGTGTTTGTGCCCTTGAGTCCTCACCCTAGCACCGTTGAATGATTTTAGGATTTTTCTATAAACTAACCCCTCCCCTTCTCTTAAG |
| Cluster-91090.1 | TTTATTGCTATTTGAACATTTCTTAAAAAAGGCATTTAATAGGCTCGGAAAAACTTATAATGGATCTTTTGGTGTTTATTATATTATTGCTAATATCCAATCCTTACTTAATGTTTGAAGGAAGTTTCAACATTTTATTCTTTTCTTAATGAAGCTCATTATCCATTACTATTTGGTCAGTAACTCCTTTGTGCACAAGAAGCCCACTTTTGCAAAATCTTAAAATGTTTTTACAATTCATGACGTCCATCCCAATATCCAAAAACCACCTTATTGCCTGCCATATTAAAATTTACTACATCTTGCTCTCTTACCGTCTTTTATTTCATTTTCACTGTCTCTTTGTCAATCTGTGCAGTTTGCTTTTGTGTGGGTAGCTGCTTATTAAATACACAGCCTTCGTGTCACTACAACCTCCTGACATTTTTTTTTACTGGTATAACTTTTTCAGATGATGGATTCGGATATTGAACCTCATCTCTCTATTCCTAAGGATGGCAAAGATGTCTCCACCCCTTCAAACTCTTCTGAATTCATTGGCATTGACTCTCTGGTCACTGACATTTTTGCTACTTCTACTCTTAATGAACTGTCAAGAGATGTTATGGATTTTGCAATGGTAGGAATTGATAACACCTTTTCCCACAACTCTATTCTATAACAGGACATGGCAGCCAATACCACCACTTTTCCTTCTATTTCCCCTTCTAACGGGAATGTGAAGATGGATTCTGTAGCCATCACTTCCAGTGGATCTTCTTTTCCACTTTTCAATTCTACTTCTCATACTTTCTCAACTCCCTTTGGCAATCCCTCCTCGCTAATTACTCCATTCTTTTCAGTTCCTATGCATGGGAGTGCTTACCAACTTCCAACCACACTTCCAACAATAGGTCTCTCGGCATCTCAATAGGAGCACCCACATCCTCTTCAGGACACAATGCAGGAACTTCCTTTTTAATTTCAGAATCCTCTTCGTCTCCCTGCTTGTAGCACATGGAACATCAGCGGCCGATGCGACAACAAGCTTCCCATCAACAAATACCTCGGGAACGTCACCATCATGTTCCAATGTTCCCAGCATCAATGCATGAGGTTTTTGCCTTTATGGATCATTCCTTGCACATGCTCAACTATGGGAATAATCAGACTTGTCTTGAGATTGACTCTATCAACAAGATGCTTAATGTCCAAATGAGGAGGATGCAGGGATACCAAAATCAGTGTCGGCTTGTATTTTGGGAAAGAGACCTTTGCATTATGATGGTATCAGGCTTCAAAGACCCCATCATGTCATTGATGCTTCAACCTCCAAAAATCATGGCTCTAATACAAACTTTAGAAGAGGAGTTGGTAATGTGACAACTCATTCTTACGACTCTCATGGAACGTTGGAGACCATTTTTGCTGCTACAGTCACTTCTTCCACACCTTCCAAACAAGCAATGGATAACGATGCCGTGGTTCCCCATGCTTCAGTTCTTCGCTCTTCTACTATGCCAAGATCTAGCAAAAATGTGAGTAAACATTTGCCTCATAAGAAGTTTCATCACAATTAGTTGCCATCATCACTTCTTTACATTTTGTGTATACGAATTTGTTAATAGCCACGCATGTTCACTTTATCCCTTTCAATTTTGTTGCTCTCATCACAACTTTTATCATTTGAACTATGGAAATTTCGAGGATACTCTTTTTGCCTCTCGACACTTTCTTATACCTTTTTTTGATTTAACTATAAAGTCCCAAATAATGTAAGTCTTCTGTATAGGTTATATCCTTGTGTCTATCTTTTGTTAGTATAATTTTTGGTGTTTCGCACGGAAAAAAAAAAAGAAAAAAAAGAAAAAAAAAACATTACTCGTCAATTCTTATTACAAGCTTCTCCGGTTTTATCATTTATTTGATCACGTTTTCCATTATAAAACGGGACTAAACTACTACTAAAACAAATGAACACTTGGGCTATTGTTCGTCAAAGTTCATTGTTTTTGTTCCTCAATTTGGGAAGCACGTAGAGATGATTCACTCGGGTCATCATGCTTTTGTCTGCGGTCATGTCTAGATCCGTGGGTTCACCTTCCACGGAGAGCTCGAAAGTGTGAAGGAGTGAACCCACAATGAGAAGTATTATTTGAAAAGCCAGCTTGTAACCGGGGCACCCTCTCCTTCCTGTACCAAATGGCAAGTTCTTAAAGTCAACTTGGGCGGTCATATCCACGTCCCCCTCGTTTGCAAACCTCTCTGGCACGTAATCTTGCGGATCCTTCCACACCATGTCGTCCATCCCCAGTGCCCACCCGTTGATAATAACCCACGTGCCCTTTGGTATGTTGTAGTTCTTAATTCTCACGTCTTCGGCGGCCACTCGCGGTGGCAGAAATGGACTCATGTTCACCCTCATCGTCTCCTTCACTGCACATTTTAGGTAGCTCATTTTAGCCACATGTGACTCCTTCACGCTCGTCAAAGCTTCATTCTCCATGATGCTAAGACACTCTGGTCTGCTCAATAGATGGCTCAGCGTCCACTCCGTGGTCACAGCGGTTGTGTGACCAGAAGACACATGAAAACTCAGCATAGTAGATCTGATCTGTTTTCTGGTAAGAGAGGAATCTTCTTTGGATAGGTCTAGAAGTCTATCAATGAAACTTTTATTTCCTCCATCAGGTATCTCCGTGGCCGCGTGTTCGTCAAGTACTACTTCTGCAAAAGAATGAAATGTATTGTAGGTTTTTCTCATTCTTTGCTTGATTCCTAAAAATGGCTCAAAGAGTTTCAACCAAGGAAAGGCGTCATACAATAATCCGCTGATGACCATCTCTACGGACTCACGAGGAAGATGGAGGAGCTCCTTGGCCAAAATGGTCGGAGTGTTTCTAGAAATGGTGGCCCAAACAATGTCATACATGGTGCTGAAAATGGCCTCACTCAGATCCACTGGGACGGTTCCCTGTTGGCTGTCGCTCCACACCTTGCGCATCATGCAGGAAACCTGTTCTTCCCTAAACTCTCTGAGAGACTCAATACTTTTTGGTGACAGCAGATTATTGACCATGACCATCCTCAATGTCCTCCATTCGTCTCCGTAGGCAGTAAATCCCATATCTTCGCCGTGGAGGAATAGGTGCTGGGCCACTGTCGACTGTGGCCTGTTTATGAACTGCATCTCGTGAGTTCGTAGCAAGAGCTTCGCCTCCTTTGCCGAAGAAACTATCACCGCATTGATGCTCCCAATAGGGAAGGCCATTATTGGCCCGTACTCTCGGGCAAGGGATGTAAGAGACTTTTGAGGTTCTTTTCCAAATAAATAAAGGCAACCGATGAAAGGCAGAGGAAGTGGTCCCGGTGGGAGTCCCCTCCTCCACCGAAACATAGAGATGTAACACGCCAGCACCGAAAATGCCACCGCCAACGCTATCTCATACCATTCCAAAATGCCCATCTTGATTGCTATTCTTTTCCACACCTCGCTACTCTTGCCGTCCATAGAGCTTTATCCCCTCTTCGAATGAGAATCTAATTGAATACATCCCACACCACGCTTCACCATGCCTC |
| Cluster-100785.2 | ATAGCAGTCCGGGCTGGGTGCAAAGAACAGCAGGGAATCACACGAGGGCGGGGTTCACGGTGTACAGACGGGGCACCCAGTGTCCGCGGACACCGATGAAACCCTGGGCAACAATATACTATGGCGGCTCTGGGCTACTAGTTGCGTTACGCCCTACCCGACATGAGTCCTCATACTCCCATGGCTGAAATTTCTCCTTTCTCTATTTTTGTCTTTCGCTTCAGATGGTGATGTTGCAAGTTCGATGTGCAGGCTTGTTGGGTCTCATGTTGCTACGTTTGGTTTGCTCAGTCCCCCAGATTTATTAAGGTTTTTTTTTCTTCTTTGGTTCTTTAATTTGTAAAGATGTATTGGTAGTATTCTACCATTTAGTCCACCTAATGGTGATGTGCCTGAATCCATTTCTCATTGGGAACATCAGTAATAATGTAGGCTAATTTCTTACAAGAATCTGAGCAATTATCTTACTGGTTCTTTCTACAATGCTAATTCAAACCCTCCCATAACTGGCTATCCTTATTAAACATACACTACGTAGCAATGTATCACAAATGAGCATAATG |
| Cluster-101463.0 | TGTGATTTCGGGGAAACCAACAATTTTAAGATTGTCGTAAGGCAAACAGAGCTGTTAAATCTTGACTGCAAAACCTCTCCCCTAAACCCCAGACACATATTTGCTGTTCAGCTGTTCTGTATTAGGGTTTCTCAGCTGTGGACAGAGCTTCTGCTAACCTGAATTTCCCACAAAGGCAAGGCAATGGCTGCCTTCAGCTCTGAAATACCAGCATCCTCCGAGATATTGACGCCGGATGCAAAAGAGGAACAAGATATAAAATATAACCGAGAAGACAATAAATCAACCATGGAAGAAGAAGAGAAAAAGGTAGCAAAGGAAGAAGCCGATGAGGAGGAAGAAGAAGAAGAGGGAGAATGCGGGTTCTGTCTGTTTATGAAGGGAGGCGGATGCAAAGAGCCCTTCGTTGCCTGGGAGAAATGCGTCGAGGAAGGCGAAGAAAAAAACGAAAACATCGCGCAGAAATGCTTCGAGATCACATCTGCACTGCAAGAATGTATGATGCAACATTCTGAATATTATAGCCCTCTCCTCAAGGCCATGGATGGTAGTCAGTCGGATCAACCCGATAACGCCGACCCATCAACCGAGACCGACAATGACGCCAACCCATCAATTGAAACCAAGAATGACGCCAACCCATCAATTGAAACCAAGAATGACGCCGACTCAGCTCAAGAAACTAAGGACCCGGTCCAGGAAACCAAGGATTCGGCTCATAAACCCGACCAAGTTCGTGAAAGCAGTGACCAAGTTCGCGATACGAAAGATAAACCAGAGGTCAGCAGCTCCAGTGATAAATCTTTATAGATAAACACGAAGCCCTTTTGTGGGTTTAAGATTCATGGGGTTTACATTGCCCTTTTCTCTTTACAGGTATTTTTTTTGACATCAATAATATCTCCTCTATCATTAGAGCGAGTTTGTTTCTAAAGTTGCGCTTATTTAATGGCCGTGGTCTATTTATGAATCCACTCTTATGAATTTCATAGCGATTGTAGTAACGAGTTCTCATTACTGTGACTAATTCGGTCAACCTGAATTTTCATAGCAAAAATGAACTATTTATGCTGCAATGAGATTCATTTAGTTAATTAGTTTTATGAATATTTGTTCATGTTACTGCTTTTCTAATGACATTTAGA |
| Cluster-3061.27 | TTCCGCTTCATTGAATAAGTAAAGAGACGATAAAGGTAGTGGTATTTCACTGGCGCCGGAGCTCCCACTTATTCTACACCCTCTATGTCTCTTCACAATGTCAAACTAGAGTCAAGCTCAACAGGGTCTTCTTTCCCCGCTGATTCTGCCAAGCCCGTTCCCTTGGCTGTGGTTTCGCTAGATAGTAGGTAGGGACAGTGGGAATCTCGTTAATCCATTCATGCGCGTCACTAATTAGATGACGAGGCATTTGGCTACCTTAAGAGAGTCATAGTTACTCCCGCCGTTTACCCGCGCTTGGTTGAATTTCTTCACTTTGACATTCAGAGCACTGGGCAGAAATCACATTGCGTCAGCATCCGTAAGGACCGTCGCAATGCTTTGTTTTAATTAAACAGTCGGATTCCCCTTGTCCGAGCCAGTTCTGAGACGGCTGTTCGTCGCCTAGGGAAAGCCCCCGAGGGAGCGCTCTGCGACCGTCGCCCTCCCGGCACGCGTAGGACCACCGTCGCCGCTGTTGCAGCTCGAGCAGGCCCACGACAGCCGGCGGGTTCGGGACGCAGATCCCTAGACCCAGCCCTCAGAGCCAATCCTTTTCCCGAAGTTACGGATCCGTTTTGCCGACTTCCCTTACCTACATTGTTCTATTGACCAGAGGCTGTTCACCTTGGAGACCTG |
| Cluster-5366.78 | GGGACCAGTGGAAGATAAATAGAATGTGGTGACTGTGCCTGCAGAGTTTCCTCCCACCAGCTTCATTCTCATGTCAAACCTTCCATGTAGATGCTCCTTCTTAGACTGGAATGCAGCACCAGAAGATTTGTCAAGAGAGAGTTGCATCTGCCTGCCATTCTCCATGATGTGAGCATGGTCTGCTCCCCATGTGATGTCAAAGTCTCTGTTGAAGTCGGCCATGACTGTGGTGCCCAATATGAACACCATGAATGAGTAAAGAATCATAATACTGTTTTTGGCAGCCATGGTTGAGGGTTTTGGGATAAGATAAGGGATTCTAAGAAGATGCAAAAGTGAGATATTGAAGAGAAATGTTGGATGCTTTTGTGTTGTATTAAAAGTATGAGCTGAGAAGTAGGGATGGCAATGGGTCGGGTATGGTCCGGGTCGACCCCTTACCATACCCATATTCGTTTATAATTACCCTTACCATACCCGCCCCATACCCTGTAAATATTAATCGGGTATTATCCATACCCGCCCCGATTAAAATACGGATTACCAAACCCGACCCATAACCGCCCCGTTTTAAAATATAATATTATTAAACAAAATAATTATGGGGTAAATTACCAATACATTAGTCCAACATGTTAGAAACTAAAAGATAACCATTTCTAAATGCACTAAATAAAAAATAATCTCAAAGTTCATGTTAATATGAAAACTTTGAATAATCCCAATGATTTATCTGTAATCCATGAGCGCTTATCTTTCGGACTCACAAGTCATCAATAGTCTCAAACCTTTCTGCAATTTTGCTATAGTGTTGTGTTGCTAGCTTGCTACCGTCACCCTTTCACTGTATTGTTGCCGTCAACCTTTCTGCTTTGTTGTTGGCTTGCTGCCATAGTGCTATCAATCTTTTAGGTCTTTTATGTGTATTGTGTAGTTGTGTAAAGTGAGAGATTGAGGATTAGGGATTGTAGAATTATGTTTACTAAATAATAAAACACATGTGACATGTGACTTATTCACCTATATAGTTTTAAGTGGTTTAATATATATATATATATATGTCTAAACGGGTAGGGGAGGGTCGGGTACCCGAATATTTTTTATGGATTACCAAACCCGACCCGATACCCGCCGGGTTTTATTTTCCAATTCCCATACCCGCCCCTAATCCGATTGAGTCACTACCAAACCCGCCCCATTAGGGTCGGGTCGGGTCGGGTACCCGCAAAACCGGGTCAAATTGCCATCCCTAGCCTCTTCGGAGCTCTGAGAGCAGCCGCCATCCATATCAATGGAGTCCGCACGGGCATGCTCGTGCCGTTGCACCGGGCTACATCCCAACTCTAAATCCACTTTACTATCCTCCATCATGACAACATATTCCTGGTTCCAGCCAATAACTTTGCCATACATGAACACAACTAAACCCAAAACAGCCGCAAAGAGCCAGCAGAACTGCAGGTTGGCCAGAGCCTTGGCTCTCATATCCCACTCATGAGACGTGCACTTGACAACGTCGTGTCCGTCAATACGGTGCATGGCGCAACCCTTCGGCACGAAGGGTTTGACCCAGAGCACAAAAGCCATCTGGATAAACCAAATTCCCTGCATAACTAAAGAGGTGCTCCTTGTTACTGCGAGGATAAAAGAGCTACGATACTTGATCTCCAACATAGCGGATATTACTGTGACCATAGCGACCAGCTGCAGGAGCCAGTGGTACTGCCCTTCCACTCCCATGTGGTCGGCAGAGTGAAGATGAAATAACAGCAGCTCAACCATGAAAGCACAAGAATATAGTGCATGGAGGGCGCCGGAAGGTAAGTGATGCTGTCTTTTATCCACATACAGAGCAAACGATGCGTACAGTAGCAGAAACATTGATATAGTGGAGTGCTCAAAGTTGTTGAGATGGTTCACTGGTATTGTGAGGTCAGTGTCCAAAGGTTGATGTTTAGCAGGTCCTATGAACAACTCTGCTGCTATTGACAGCAGAGAACCTGCCATGAAAATTATGAGCTCTGCACATCTTATATGGCCGGAGGATGAATCAACAGGAAACCATGTCCTCGTGCGGAAGTTGTAAGGCGACTTGGCGTAGTTTTTCATTGTGTTATAGAGATGCCAAATCCCTATCATAAGGAAGCCTGCTCCTGGCAAAACATGACCAATAAAAGTGCCCATATTTAATGGTAAATGACTTGCAATACGTTTCTAGGATGCTCTAATGGCAAAGAACTTAAGAAAATGTGAAATGATGGAGAAATTCTTGTCTTTGTCGGGGGAACTGTTGGAGGGATTTATGAGTGATCGAGAACTTG |
| Cluster-14810.0 | TTTAAGAATAAACTTTGTGAAATTTTTGCTGCTATATCTTAACATTAGTAGAAATTTGTACTCCTTAGAGTCAATAAAGGGATATCCAAACCCACATGGAAAAAAATACATATATCGATATAAGTTGACTATTACAAATAACCCCTTCCGGATCGGTGAGCACCAACATGGCTTTTCCTGAAATAGTCGGCATTAGCCTTACCATCTGCTCTGTTCTGAGTAGCTATCCGCCATCCTTTCCCTGCACCCCAGCCAATTTGATAATCGCCTGGCTGACAAAATTGCTTAGCGCAGACCCTTCTCTTTGCTAGTCCTGACCTAGTCGTCATTACTGATGCAACTCCGCACCGTCGCCCACCAATGAACTCTGCCACGGCCTAGAGAAGCCTTGCCATGGATCACCCTTTGCCTCTCCATCTCAGCCATCTCGTGCATGGACTCCTCCTTTTGTTAAGCGCATCCCTGCCTGCAGTCATTTTTGTGCATCTGCAGCTTCCCTCCGCCCTCTGCCTCATCGGACCCTGCAAAACAAGAAAGGCCCTTGAGACCCTTGCCCCATAGCTGCCAGTCATAGCCTGTGCTGCCATCCTAAACCCCACATCCTTGCCTCCTCTGAAACTGCCCTTCTCCCAGCATCGAGTCTCTGCTATTACGTCTCTCTCTCGTGTTTCCCACCATCATGCCCACAGTTCTCTGTCGATCCCTGCCTCCAAGTCCTCACAGACCCCGTCCTTCCAAAGATCTGGCTTGGGCCAAGAACTTTCCTTTCTTATCAACACCGTCTCTCTCGTCTAAGACTCAAACAAGCACTACCGATCTGACCTTTCCCTTGCTACCAGGTATATGAATCTTTATGCCGTGACCTCCCCGGATAGCCGCCACTGCTTCAAAATTCTCAACCCTAGCTCCGCCGCTAGGGGTTTCCTTCTCTCAAAGCTCATAGCAAAACATTTTCCTTCCCTGACTGTTAAAAAAAACTCCAGGAAACCCTATCACTAAGCCACAATAACACTCCCCTTCTAAGTTTCTACTCTAAATACCATCCCTATACTATGTCAAAAAGAGGTCCTAAGACAGCC |
| Cluster-103987.2 | TTTTTTTTTATTAAAAAAGGATTTTTGTTTTATTTTTACAATCTCCTGTTTTTGAAAGGTAATACCCTGTCAAACTCACTAAATTTCCCAGAAACCTAAGGTAATACCCTATCAAACTCGGTAAATTTCCAAGAAACCAAGGTTACTGGTTTGGACAGGGATGACTGTACCAATCTTGAAAACGTAATTTTCCATCTCATCAATACCATGTCTTCCGTATATGAGTACGCTAGGCACTTTCCAACGGAAGAAAAGAGTTAAGATAGTCAAGTGAAAAATCTAACGATAAGAAAAAGGAAAAAGCATATTACAACACTCTACTCAAGTACCACATGAGAAAATGAATAGCTGCCTCCTATAGACATTTTTTATAAACAATACAATTGAGATACAAACAAATATATTAATATCAGAGCAGACACGTGAGCATCAACCACCTTTTAGAAACCATTGGAAGGGCTACCAAAACTTGCTCTACATGCTGCTAATGCTCAATGAGCATCTGTAATAACTTGAACATGGTTCGTTCTTCAACACTCTCGACCATTTTCATGTCTGATACATGAGTTACAAAGGCATCATCTAGCATCCATGACAAAAATATAGATAGTTTTAAATATTTACTAGAAATTCATTTACCAACAACAGAGGCTCAATGATGTGAATCAGCTGAATCTTGACTCGGAATTCTCTTCTTATCTCTCCAATTTTCTCCCCACATTTTTGCCTCTGCAAGCGCTCGCTCACGATCTACTGAGGATCTTGAACCTTTTCCTGCACTTCCAGGGCTATCCGTAGAAGCTTCGTATCTCCCAGAACCTCCTGCCAAGTTGAAGTCATGAGCAACTTTTACATGCATTGCATTTAATAGCCCATTTGTCGCTTTCTTTTCAGAATCAGATGGTCTATTATCAGGGGAGTGTTGCATTGGCATCAGTAAGGCCTTTGTATCTCCACGTTTGCTTGCTTTCTCTTCTGGCGTTTTCTTTTCTTCCCTTTTGTTAGAGGTGGCGGAAGAGCCCAATCGAGTACTCTGAATAGGATGCGTAGCAGGATCATAGGTTTGTGATGCGAGATAATTAAGAGCAGTTACAACATCAGCAATCAGCGGACGGGTAGCTGCTTCCTCCTGCAAGCACATCGCTGCCACAGCCAATGCTTGATAAAGGCCTCTCATTGGATAACGTCCTTGAAGTAACGGATCAGCCATTTGTGCAAACTTCCGCCTGTCTTTGAATAAGGGACGTGCCCACGCAACCAGGTTGTGCTCACCAGCTGACCGTGTATTATCAATAGCTTTCCTGCCAGTAATTATTTCCAAAAGGACAACCCCAAAGCTGTAGACATCTGATTTTATAGTTAGCTGGCCAGTCATGGCATATTCCGGAGCACAATATCCATATGTTCCCATGACACGTGTTGACACATGAGTTTTATCACCAACAGGGCCAAGTTTGGCAAGTCCAAAATCTGATAACTTTGGATGAAACCCCTCATCCAACAGGATATTAGATGACTTTAGGTCTCTGTATATAACTGGTGGATTGGCTCTATCATGTAAATACTCTAAGCCTTTTGCAGCACCTTCTGCTATTTTCATCCTGGTGCACCAATCTAATGGAATTTTCTGAGGTGAGAGATCATGCAGATGATCCTCCAGTGAACCCAAAGGCATGTATTCATATACCAATAACCTTTGGTCTCCATCGGCACAATATCCAATAAGGTTCACCAAGTTGGGATGATGGAGAAGACTAAGCATCAAAACCTCAACCAGGAACTCCCTATTACCTTGCAAGCCATTTCTATCTAGTTGTTTGACAGCTACAACCTGTCCATTTTCAAGCCTTCCTTTGTAAACTCGACCGAAGCCACCTTCACCTATTAAACACTCGGGTCTGAAATTTCTTGTTGCAATTGCCAGCTCACGGAAAGTGAATGTTTGGGCTGCAATATGTTCAGTTCCATCTTTAGAAGGATCTTTTCTGTTATCACTAGAAGGCCTCGCGGATTTCAGCTTATCATTTGGCAACAACACGGTCTAAAATTTCAGAGATGTAAAACTTCGAGGAGATAAAATAGTTAACCACTGCAGTTTCTTATTAGTAACCAGGTCTCGAAACAGACTGATTATCACCAGCAGTCGCAGCACGAGAAGAAGAGTTAACAATAGGGGGTGAGGAGGAGTTGGACTTCTTGTCCTTGCGATTCCCATAGCAAGGAAAACAGATGTTCATCTCTCTCTCTGTTTTCACCAAATGCAATCATACAATCACATACTCCTCCACTTCTCAATAACTCCATCCATAATATCACAGAACCTACCCAAAATTCACTAAAAAATCGAAAAATCAGTAAAAAAAGCTGAGAACAAATTCAGGAATATAAACGCGAAGCCGATCCAATACAAACCCTAGCTGATCTCAGTCTCCATCAGATCCTACAAGAACTGAAGAAAAAGTGCAGATGATGAACTAGGTTTTAAATTCTGAATTTC |
| Cluster-6901.2 | TTTTAAGGACGTGATAACCACAATAAGCAAGCTGAGAAGTTCTATGTTTTTCTATTATATCTTTACATAAATTTATAGACTTTCTGTTTTTAAAACAACATTTTAGAAGTCACTTTCACCAAGGCTTCTTTCCAAGCATAAACCAGCAATGTAAAAGCTTTCATATTTCCAAATTACTACAAATTAGCAGTAATTTAATACTAACAACAAAAGAGCTAAATGTGATTTCATTTCACTAATGCTTCCTAGTAAACATGACCCTTCTAAGCATAATTTAGTATGTATTTGGAATCCTGCTATTTCTCACACGCAAGTAATAAAACTCAGCTCTACAAAACTCCATGAATCAGCAAGGGAAAGTTTAAAGATATAAACAAGCTTTCAATTCCAATGGATTCAGTCTTCATCAATCTCGGTGCAATCTGCAACCATTTGAGCTTGTGATGATTGATTTCCTCGCACGCGTAACTCTGCAATTTCCATTATGAGCATAGGATCACAAACGAGCTTCTCATATGTGACAAACTTTGGACATCCTGCAGCTGCACATACACGAGTACGTTTCTTTCCAGTAACCTTAATGTGCTGAATAATAGCTTGTTTTTCATATATATGCCCACAGTCGGTGCTGCGAACGGGCTCTTCAAGTTCAGTGATATGCTTTCCGGTCAACGGGCAAGTAGTGTTCAAGAGATTGGATTGAGTACTTGTCATAATAATGTCCTCCTGCTCCTCTCCTGGCATAGGCTCCCCTGCATGATGAACTTTCCAAATAGCTTCCTTAAACTGTTTGTAGAAAACATGGTTTTCAGGAGCGGAATCAGACTGATTTTCAAGATTAGATATATGATCATCTATAAGCTTTTTAAAATCTGTAGACTCATTTGATGGATCATAATTTTGACCTATCGTCTGTAATGCCGAATTGAACCTCGTGATTTCATCAGTGAGAGGGATCAGTTCTAAGGTCGCTGCGTCCAATTTATTCACCAGATCAGGACAATTGTCTCTCTCTAGATCTTCGGCAACCATTTTTAATGCCACTAATGTGTTCCTGACATCTGCCAAGAGCAAATTGTTTGACGCCTCCATACTTTTAACACTGGAGCCAATTTTCTCTGCCCTGGACATCTTTGCTTACAAATTCAAAACTCTCCTGCAATCTGTATGTAGAATTTGGAATGTGACTGAGGGTGGGAGGTTGCCCTTGCCCTTTTTTTTTTTTTTTTTTTTTTTGTAAAATCGCCAGAATTTTATTAATAGTAAGTAAAACTATACATGATAGCAAAAAGTACAAAGTATGCTTAGATGGCTGACATTAAGTATACAGAAGAGGACTGAAGTAGTAGACGTAGGAAGACGGTTTTGTAGGACTTCGCAATTATACATGACCGGGAGACAGCTGTTTTACACAAAGTGACCAAAGGTATTTACATAAAGTGACCAGTAAGAGTTATTACAACATTTAAGACTTGGAGGTGGTAGACTTAAAAGGACGTGGTGGTGGAAGGATGCCGGCTCCAAGATTTGAAGACTGCCCCGCAAATCGTCGGCTACTAGAGGCATATGGAGGTGTAGTCCTGGCTGCCTTCTTAAGCTTTTCAACCCTTCGAGATGTGGAAGGGTAAAAAACCGGTTCCGGTTGAGGTGAAGATAAAGATCTGGGTGGAGATGATGGGGGGACCTGTGCAGAGAGACTCGGAATAATGCCTGTCATCAAACCCGCATTATTGATTATAGTGTTGGATTCATGCT |
| Cluster-84327.3 | CATCCTAGTTACATATTTTCTCCTATAAAGCCATCAGTCTTAATTTCTTTTAGATTAATTATTTGCTACCATCCTAGTTACATATTTTCTCCTATAAAGCCATCAGTCTTAATTTCTTTTAGATTAATTATTTGCTACCATCCTAGTTACATATTTTCTCCCATAAAGCCATCAGTATGATTTCTATGGCGCGGTGCAATTTGATAGTGAAAATTCCAAATGATTTGTATAAGAGCTTCATAACTTTGACATTCATGTCATTTGCTTTTTTTTCTTATTGTTTATAGAAATTTAGCTTTTGTCCAACCTTTTTTAAACACTGATCAGGTTTAACTTCGACATATAGATTTAACAAATATTCTTAAAAGCAGGGGCAGGAAAGATCTTATAGTGTTTTTTCTGTTATCTTTTTAAACAAAAGACGTCAAATGATTCTTTATCAGAGTTACAGGCCTTGTGGACTTTTGCTTCTTTGTTCTTATATTCAACTGATACAGCATCTGAGCTACAGAATACAGATCATAATAGACGATGGTCTCTAGCTATCATCCTCTGGCTGACTTTGGAAGTCTTCTGTTTCTCTAGTTACAGTGTCACTGTATTGTTTCAACTTAGGTTTCTAAAAGGTGCATAATTATTGGTTAAACTCCAATAGGACCAATGTAAAATATGAAAAGGTAGTAAGGTACTATTCATTTCTCTGCATATTTGATGTACGACTTTGAAACCTAATTAATTTGAAGCATTTCCTGTGACCTATAAAATTTCAGCTATTAGAATGCATTTAACTCTGTTGGTTACAGTCTAATATATGAATCATTGTTAAATATACTCTCTTTATGTTGCATTTTTCATTGTCATGTCTTTTCCTTTCCAAAATAAGGTTCTGTATTTTTTACAAATTGGAAGGTAACATGCTCAATTTGGATATGAAATGCCAATGTGACTTCGGATCGAGTATAGCCTTCATGTCATAAGTTCGTTTTCCTTGAGAGGTTATTGAGATATTTCCAACTTTTTGTAGGTTAAGAGGCATCCATTTTTCAAAGACATCAATTGGGATACTCTACAGCGACAGAAGGCAGCTTTTGTACCAACATCAGATGGGGCACTTGATACTAGTTATTTTACATCTAGGTATGTATGGAACTCGTCAGAAGGAAATGTATATACAAATGATGATTTTGAAGATGTAAGCGATTCAGGCAGCTCAAGTGGAAGTAGTGGTAGTTTAATCAATGGACGGGAGGAAGTGACGGATGAATGTGGAGGGTTAGCCAATTTTGATCCTACAGCTTCTGTAAAGTATTCTTTCAGTAACTTCTCATTCAAAAACTTGTCGCAGTTAGCATCAATCAACTATGATTTGTTAACCATAAGCGGAAAGGATTCTCCGAAGGCCAATGATTCAAATCCCGCATTTTCTTAAATATTGATGATGTTGAAGCATCATAAGTGAACCCAGGCTTTATGGAGATGTAAGGTAATGTCAATCACTAATTTTTCTGGATTGTGATTAAATCCACTTCCAGCTGTTTCTTCTGAGCGTCAGATCCTTTGTTTCTGCTACTTAAAGATGTCCTTTTATGGACTACAACATTGGTTCGAGTGCAAGTACGAAATGACTCATTGATGGTGGTAATGTATGCTAAGGATTGCGGCAGAGTATCGTTGCTCAGAGTGTCATTTTTGACAATGCTTCCTTTCAATTACAGTGTCTGTTTATGCTGTAAATTGCCTTCCATTTTAAATGGGGTTTCCTTGAATCTGTTATATCCTGGCACAAGATTTTATATCTGAAAATTACATTTGTGAATATAGTAATTGGGTCAATGTAGCGATTGAATGGCAAAAGTGGACAGCATCTGTTTATTCGAGCTAAAAGGCATATTCAATCATGCACAAGAAGAGAATTTTTCAAGTATTTTTCTAAAAACTATGAAGAGATGTATCCAATGTTGCTGTGTATCATTGGTTTTCCTATTGGCTTCCAACACATTAATTCTTGATTCCAACAGGTAGGGTTATTATTTGACCTTCAAAATTGAGAAAGCCTTTTTGATGTGATGATCTATGAAATAAATGCCGTTACGTTGAGTCTCTGTCAGATAACATGGT |
| Cluster-94048.6 | GCAACATCTCATGGTGCCTACAAATAAGAACTATGTCTTCTCGTTTCTCGTCAAACCATAGAGCTGCTTAGTATTATCAGTAAAGAAAGTGCTGAGTCGCTTTCTCTTTGACAAGCTATGAAGATGCGATGCGGAGTTGTGGTAATGTTGGTGGTGTTGGTAGCTATGGCCGTGCTCGTACCAGCAAGGCATGTAAAGATGGAAGGAGATGAAAAACGAGTACTCATAGGCGGGGTAGGAGGAGCCAGCAACGGTGCCAAGATAGCGTATGGAGTACAGAGTGTCGGTGGATATGCGAAACAAGGTGCCGACGGTGTCAAGGACCGCAAGGTTGACGGGCGTGTGAGGCCTAACGACGACGATGACGACGACGACGACGACGATGACAGTGACGACCGAGACCCAGACCCAGACCGAGACCGAGATGGTGACGGAAGGCATTAAAGCGTTACATCCTGAGTCCTTCACTAGCTATTATGGTTTATGATTATCTCATAGGCTTTGCCATATGCTAACTACTTAAAATATATATATCAGTAGCTATTGTATACTAAAGTTGGTGAGTGTCTTAAGACACATACATGTTTTACTATTGTGCATCCTAGTTTTTGTTTATGGTGAAGTGATGATGAACCCTGCACATGTTTTCTCTGTTATGCATCTTAAGCTTCTTAGCTTTTGATCTGTAGTGGTGAGTGGTAGTGGCCTCTACACCAATATTCTAGTAATAAAATTATTATTATATTTACTTTGTAAAACAAGGTCTTTGGCTGAATTTGCAACGCTCTTACAACTTCAGCATCTATTTGAAAAATATCAAAGAGATGATCTATATTCTTAAATTCTATGTGCAAAGAACACAATTCAGTCGGAATTGAGTTTAACCTTAGACAAGTCATAAGAAGAATAAATGAAGACAAAGTCTTAAAGTATTGCAAGACTTTAATTTAT |
| Cluster-57672.12 | ATCAACAAATCAACTTTATTTGAAATTTCGGGGACGAAATTTATTTTAAGTGGGGAAGAGTGTAACGACCCTAAATTTTAGGATCGTTAGTGGAAATATTAGGAAGGTGAAAGATCGAAAATCGAGATGATGTACCTAGTTAAAACTTGCCAGTAGGGTATGTATTCAGCTTTTGTATAAAAAGGGGTGTATTTATCAAAGCTCTTTTAGTGGTGGGTGTATATAGTGAGGGGTGCCACTATTAAGAGTGGGGGATGTACTTAGAAAAGGGGAAATGTTTAGTCCCACATTGGTGAGGGGAAGTGAGGAGCTTGAGCCAACTAGTATAAATAGGGGATGAGGGCTATGAAACCAAGCACACCTCTCGCCTCTCTTCCCTTTCTCTCCCCTTCAAATGGAAGGAGTTTTAGGGAATCCTCGAGGCGGGATAATTTTCTTCCAGCGCCGAAGGCCTGCCGAAATGAGTCGGGGTCTTCTCTTACAACGCCGGAGGACTGCCGAGCAAGCCGGGGAGTTATGCTTCGGCTATGGTGTCGGGAATCAAAAAGTTAAGCGCGTCTCGATTGGAGCTGGGAGCGGTTACCTCCCGGGAAGTCCTAGCGCGTCGCTCGTTCTATCGAACAAGGCTTCGATATCGGTCACTAAGCTGGTTACGATGATATAAGACTGAAGATCCTATCCGCTACTGCTTGGACGATGAAGTACGTGCTTCAGAATACATGTAGCTTCCGCTGTGATAAATCGGGTATTCAACTTTTATTTGTATTTATAGTCTTATTATTTGATCATAAATGTGTTTTGATATTCAATGCCTCCGGGTTAGACTCGGAGATGGTAACGAAACCGGGTTTTAGAAATCCGGGTCGTTACAGTTGGTATCAGAGCCTAGGGTTGAGCAAATGCGATTA |
| Cluster-76533.2 | GAAACAAGATGGCAATAATCATTAAACAAGAATAAAAACTAGAAAACTACAATGATTTAAAAAAATGCAAAGCAAAATTTCAGTACTTTTCATGTAGCTGAAAATAAGAGAACACATGTTCTCTTAATTTGGTAAAAAGAATTCAAGCAAGTTTCCTATTCCATAATCAGATCTAAAAATAAAACAGAAAGATTTTATCATGGAAAAAGCTATGTATGTTATTACGGGTTATGATAGTAAAATGAGGTTCAAGAAAATGAGTACCAATATTCCAAATTTTCTCTGTGGTCAATCATCATCTACAACCTCTTTCAAATTACACAAGATTAGCAATTTAGAAAAGCTTATGCTTCTTGCATGAAATATGGAGCACAGACTTTGTCATAACCCTGGTTTGGCCATTTTGGGGGTTTATAGATTTAGAATGTATCATTTGTGGTCATCACCATCTGCAGCTCTATTAACTAAAAAAAATTACTAGTAACTTTGCATTTTTTATGCTTCACAGCATGAGATACCAAGTAGACTGTATTATACTCTTCATTTTGTCATCGAAAGCAGTACTAATCTTATTCTTTTGGAAAAGCTTTTATTTAGTTCACTTTTCTAGCTTTTTTATCTAATTCCCTAATCTTAGTTTTATTTCTTGGAAATGATACAAAGTTACAAACTCATATACTGATCAAAATAATCACATGAGGCAGATACATACAAACCACACCAAATTATATAGACCTAAATACTTACAAGGCATACAAAAATCTAAACATTAAAGAGTGAAATTTAAAAGTTCAAAAAATTACAGAACGAGTTATACTGAAATGAAAGAAACTTACATCCTCTACAATGAGCGATAGATTGGATTTGACAGAAAGAACCATGATCCATTTAATAAAAAAAACTATTTGTGTCAAATTCGCTAAAGATATCATGTAGTGTGTTATGCCATTTAATTTGATGTTTGTTAAGCATTTTGCGTATACAAAGAAGGCTGTACACTGTTAAGACTTCTTTGCCAATGCAAAATATGCAAAATCTATCATCCTACATAAATGACAGAGAATACAATGAAATTTCATCACTCTTCTTTTCTCAAACCTATAGATGCCTCTCTTTTATGGAGGATGCTGCCAGAACAAGGGTTTTACTAAATATAGATTCTGTTCCTTGTTGCCTCTGTGTTCTCAAGTGATAATCCGTAAAGCTATCTCTTACAACATAAAGTTGTTGTATTGCCCATTATCGCTTCTTTTAAGATCTGAGGAAGCGATGCTAAGTCCACAGGTGGAAGTTCTCTAAAGTCATTTCCCATTCCATGAAAACCTTCATCATTACCATGAGTCGCTCCAGAATGCTCA |
| Cluster-90110.3 | CGAAATTGTGATTGGGCCTGCACCTGTATCACAACAATTTATATTCATATACAACTTCTACAGTCTTTCCTAAGTGATGGCACTACTGCTATACCACATCTTTTTTCCATGTTTGTTGAGCATATCTAACAGCAGCAAGAACTGCCACAACAAAGTGAACCATTAAGAAGAGGCATAAGCTATAGAATCACTGCTTAGTAAGAACAGAAACATAGCACATTTAAGAGGCTACCAATGTTTACATCACGCAAGGTAAAATCTAAAATACTTAACATTCAATAATGGGTGAAAATTCCCAATTTGATTTTGTTCATAAAAGAGAGCTTTTGGTTGCTGATATTTTTTTTGTCTTCTAGATCTACACCTATAATTGAATTCAAAGAGGATCTTTGAAATCAATACATAAATAGAGTTTCGAATGAATATATGAGGCTATTTCTATTGATGTTTTAGTGAAGATATATTGATAGACAAAAAGGACTCTAGCACAAACAATAAGGTGAAGCTATTCCTTTGAGGTAGCTCCAAGTTAAAAGCAACAAAACTAAACCAGAGATCATGTGGTCATGATTTTTCTTAGCATAAATAAAAATGAGTCAATTTCCAAAATACTATCAGAAAGAAGGATGTATCATATTGGAAGGAACACATTGACAATTTCTCCAGTGATTGGCATGAAGGCGAAGAACCTCCTTAGCCTTGTGCTTGGCCTTGAGAGAGCATTCCATCTGCAAAACCATGCAGAGCTTCGAGACAGCTCCACACTGTGCCATTTCACTCAACAATCTTCCATTTGGGGAAAACTTGCATAGAGACCAGAGAACTCTCACCCCCTTTTCAGTAACAAAATGCGAGACTCTAAGAATCTTCTTAGAGACCACTGCAACAGTCAAGGCATGGCCAAGGGCGACAGCGCGCCCTTCGGGGCACTCACACAGAATGTCCAACAAAAGGATGAGTTTCTCACACACACTTCTCCTTTGTTGAGGTTCAGCCAAGAGTTCCAAGGTTGCATATAGAGCCCCTGCCTCAATTGCTATGACTCTATTCCTTTTGTTATTGCTACAAATAGCACATAATACTTCAATCCCTGAGGATGAATTTTCATCTGCAGCCAATTCTACTAGCCCCTCTATAAGATCCTGAGTTGCATTTGCTATTACATCTTCTCTAGCTTTTCTGCAACTTGACATTATGATATTTCTCAGCACTGAAGCCACATGGAACCTAGCTCCACAGGTGCCTCTCTTGAGTATCCACGCCGCGGGCCTAACCAGCTTCGCGGCCACCGCTTCGAGGGAAGAAGTATCTTGTGTTATAGTAGTCTGATCTTGTTGAAGTTTTGATATGATTTCTAGAACCAACTCACATACAATTTTTGCATCATGACATCCACCTTCCATCTCTTGCAAATCTATGAAGGACACCAGGGACTCGAGCGACATGAACTGGTGGAACAAATCCTTGTGCCTGTGAGTTCCCATGAGGGCTTTGAGATTCCTCATGGGAACTATCTTCATGCTTGGGGAATTGCATTTACGCAATTCCTCGAGTATCTCTTTGATTTGTTTAGGATGGAAGGCATTGAAATCAGTATGTGAAGCAGAGGCAGAAGATGATGATGAGCCTGTAGTAGTCATCCATGACTGAATTAGCCTTTTGAGAGTATGATTTGGGGTAAGGTCTTGATTTTCGAGTACTTGCATAGTCATCGGACATGTCCGCTTCTCGCAATCCAATATCCATCTCTCTATGTTTTCCCTCTCGTAGGTAATCCCTGTGGAGACCGTCACAGGGTCATTCATAACTTGCAGAGATATAGGACACACAAAGTAAGTAGGAGGTGAAGACATTTGGTTATCTTGTGATGAAGGAAATAGTGGAATTGTAGAAGAAGAAGAAGAGTCCATCATCATAGTCATTCTACACCTACCTACAAGAAAAGATAGATATACTCTTGCAATGCTCAGTGCTCTAGATGTTGTTCTTATAGGACAAGGAAAACACAAAGGCACTAAGAAACAAAGATAACAAGGAAGAAAACAATTATATGTGAGGTATTAACTCTCCATGATTGATATGAGGAAGACAATGACAAAGGTATACTTATGCAGTTTCTGCAAAGAAACCGAGAATGGAATCTCATAAATCCAATCCCTGTGATTCTGCTGTTAC |
